# TRAP1 enhances Warburg metabolism through modulation of PFK1 expression/activity and favors resistance to EGFR inhibitors in human colorectal carcinomas

**DOI:** 10.1002/1878-0261.12814

**Published:** 2020-10-30

**Authors:** Francesca Maddalena, Valentina Condelli, Danilo Swann Matassa, Consiglia Pacelli, Rosella Scrima, Giacomo Lettini, Valeria Li Bergolis, Michele Pietrafesa, Fabiana Crispo, Annamaria Piscazzi, Giovanni Storto, Nazzareno Capitanio, Franca Esposito, Matteo Landriscina

**Affiliations:** ^1^ Laboratory of Pre‐Clinical and Translational Research IRCCS Referral Cancer Center of Basilicata Rionero in Vulture Italy; ^2^ Department of Molecular Medicine and Medical Biotechnology University of Naples Federico II Italy; ^3^ Department of Clinical and Experimental Medicine University of Foggia Italy; ^4^ Medical Oncology Unit Department of Medical and Surgical Sciences University of Foggia Italy; ^5^ Nuclear Medicine Unit IRCCS Referral Cancer Center of Basilicata Rionero in Vulture Italy

**Keywords:** cetuximab, glycolysis, oxidative phosphorylation, phosphofructokinase 1, TRAP1

## Abstract

Metabolic rewiring is a mechanism of adaptation to unfavorable environmental conditions and tumor progression. TRAP1 is an HSP90 molecular chaperone upregulated in human colorectal carcinomas (CRCs) and responsible for downregulation of oxidative phosphorylation (OXPHOS) and adaptation to metabolic stress. The mechanism by which TRAP1 regulates glycolytic metabolism and the relevance of this regulation in resistance to EGFR inhibitors were investigated in patient‐derived CRC spheres, human CRC cells, samples, and patients. A linear correlation was observed between TRAP1 levels and ^18^F‐fluoro‐2‐deoxy‐glucose (^18^F‐FDG) uptake upon PET scan or GLUT1 expression in human CRCs. Consistently, TRAP1 enhances GLUT1 expression, glucose uptake, and lactate production and downregulates OXPHOS in CRC patient‐derived spheroids and cell lines. Mechanistically, TRAP1 maximizes lactate production to balance low OXPHOS through the regulation of the glycolytic enzyme phosphofructokinase‐1 (PFK1); this depends on the interaction between TRAP1 and PFK1, which favors PFK1 glycolytic activity and prevents its ubiquitination/degradation. By contrast, TRAP1/PFK1 interaction is lost in conditions of enhanced OXPHOS, which results in loss of TRAP1 regulation of PFK1 activity and lactate production. Notably, TRAP1 regulation of glycolysis is involved in resistance of RAS‐wild‐type CRCs to EGFR monoclonals. Indeed, either TRAP1 upregulation or high glycolytic metabolism impairs cetuximab activity *in vitro*, whereas TRAP1 targeting and/or inhibition of glycolytic pathway enhances cell response to cetuximab. Finally, a linear correlation between ^18^F‐FDG PET uptake and poor response to cetuximab in first‐line therapy in human metastatic CRCs was observed. These results suggest that TRAP1 is a key determinant of CRC metabolic rewiring and favors resistance to EGFR inhibitors through regulation of glycolytic metabolism.

## Graphical abstract

Here, we show that TRAP1 modulates glycolytic metabolism by regulating PFK1 activity/stability. In a high TRAP1 background, TRAP1 inhibits cellular respiration and interacts with PFK1 on the ER and this enables PFK1 glycolytic activity preventing its ubiquitination/degradation. In a low TRAP1 background, cellular respiration is upregulated and PFK1 activity reduced due to increased ubiquitination/degradation and this results in loss of TRAP1 control on glycolytic cascade. The increased levels of citrate, observed in conditions of enhanced cellular respiration, are responsible for the inhibition of PFK1 activity, and this results in enhancement of PFK1 ubiquitination/degradation.

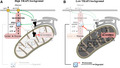

**Abbreviations**
^18^F‐FDG: ^18^F‐fluoro‐2‐deoxy‐glucose
2‐DG: 2‐deoxy‐glucose
BRAF: v‐raf murine sarcoma viral oncogene homolog B
CRC: human colorectal carcinoma
ECAR: extracellular acidification rate
EGFR: epidermal growth factor receptor
GLUT1: glucose transporter member 1
HKII: hexokinase II
HSP90: heat‐shock protein 90
MCT4: solute carrier family 16 (monocarboxylic acid transporters), member 3
OCR: oxygen consumption rates
OXPHOS: oxidative phosphorylation
PET: positron emission tomography
PFK1: phosphofructokinase 1
RAS: Kirsten rat sarcoma viral oncogene homolog
SUVmax: maximum standardized uptake value
TRAP1: TNF receptor‐associated protein 1

## 1 Introduction

Cancer cells adapt their metabolism to extracellular environment to meet the high energetic demand induced by rapid cell proliferation, promote survival, and favor long‐term maintenance [1]. In such a context, Warburg metabolism, with glucose being converted to lactate even in the presence of oxygen, is considered a hallmark of cancer [2], due to its relevance in supplying cancer cells with precursors of proteins, lipids, amino acids, and nucleic acids to build cellular structures and maintain high proliferation rates [3]. However, recent evidence suggests that (a) mitochondria still play important roles in bioenergetics in cancer cells [4], (b) specific cancer types are characterized by a prevalent oxidative metabolism [5], and (c) metabolic rewiring from aerobic glycolysis to oxidative phosphorylation (OXPHOS) and *vice versa* is responsible for driving cancer progression [6]. Intriguingly, it has been suggested that metabolic alterations sustain almost all known hallmarks of cancer and that metabolic pathways may provide very interesting and novel therapeutic targets [7].

In such a context, increasing roles are attributed to molecular chaperones, which are not just multifunctional proteins, but rather molecular hubs connecting different metabolic pathways [8]. The mitochondrial HSP90 chaperone family member, TRAP1, is involved in several functions of cancer cells and, among others, regulation of cell bioenergetics [9, 10, 11, 12, 13, 14]. Indeed, the primary TRAP1 function in cancer is still controversial, with the majority of authors hypothesizing an oncogenic role based on its upregulation in several human malignancies (i.e., colorectal, breast, prostate, nasopharyngeal, and lung carcinomas), whereas others suggesting an oncosuppressive function due to its downregulation in selective tumors (i.e., ovarian, renal, and cervical carcinomas) along with tumor progression [5, 9]. Noteworthy, TRAP1 expression inversely correlates with the activity of succinate dehydrogenase and cytochrome *c* oxidase, with consequent downregulation of OXPHOS in human malignancies with high TRAP1 expression [15, 16] and upregulation of oxidative metabolism in tumors with low TRAP1 background [14]. Consistently, high TRAP1 expression may favor glycolytic metabolism through modulation of hexokinase activity [17]. Altogether, these pieces of evidence suggest that TRAP1 role in bioenergetics is cell‐ and context‐dependent and that cancer cells up/downregulate TRAP1 expression to adapt their bioenergetics to energy requirements and environmental conditions [5]. To further characterize mechanisms involved in the regulation of glycolysis by TRAP1, this study addresses the mechanisms of TRAP1 regulation of glycolytic pathway, showing for the first time a new interaction between TRAP1 and the most relevant glycolytic enzyme PFK1 and the relationship between TRAP1 glycolytic regulation and its control of mitochondrial respiration in colorectal carcinoma cells (CRCs). Remarkably, the impact of this regulation in modulating cancer cell response to EGFR inhibitors is shown. This issue is extremely relevant in a clinical perspective, since TRAP1 is upregulated in colorectal carcinogenesis at the transition between low‐ and high‐grade adenomas and in about 60% of human CRCs with parallel overexpression of its protein network [18] and this favors a drug‐resistant phenotype [19, 20]. Intriguingly, the co‐upregulation of TRAP1 and 6‐proteins of TRAP1 protein network identifies a subgroup of metastatic CRCs (mCRCs) with poor outcome [18].

## 2 Materials and methods

### 2.1 Tumor specimens, clinical data, chemicals, patient‐derived spheroids, and cell cultures

Specimens from 26 human CRCs and corresponding normal, non‐infiltrated peritumoral mucosa were obtained from the IRCCS‐CROB Tissue Biobank (Cohort 1). Tumors were staged according to TNM classification system [21] and were selected for having been evaluated with ^18^F‐fluoro‐2‐deoxy‐glucose (^18^F‐FDG) positron emission tomography (PET) imaging before surgical removal of primary tumors. The maximum standardized uptake values (SUVmax) body weight corrected were determined in primary tumors using vendor‐provided software (Volumetrix for PETCT; GE Healthcare, Waukesha, WI, USA) and further used to establish statistical correlations with molecular data. Patient's characteristics are reported in Table S1 (Cohort 1).

An additional cohort of 15 RAS‐wild‐type mCRCs treated with first‐line standard doublet chemotherapy (FOLFIRI or FOLFOX regimens) combined with cetuximab was selected to study the relationship between glycolytic metabolism and response to cetuximab (Table S1, Cohort 2). Patients were deemed eligible if they met the following requirements: (a) primary surgery without neo‐adjuvant chemo‐radiotherapy; (b) ^18^F‐FDG PET Scan before first‐line therapy, and (c) follow‐up CT scan within 3 months from beginning of first‐line therapy to assess tumor response. Using the above criteria, 25 target lesions were evaluated. Imaging studies were interpreted by two experienced nuclear medicine physicians. The mass sizes were visually estimated and measured for minimum and maximum diameters by using vendor‐provided based software both on aggregated initial PET/CT and on subsequent CT scan. Target lesions were defined as identifiable masses/structural lesions or lymph nodes larger than 1 cm in minimum diameter with soft‐tissue/abdominal window settings (i.e., one lymph node > 1 cm or three lymph nodes in cluster) matching with a significant ^18^F‐FDG uptake on PET scan. The maximum standardized uptake values (SUVmax) body weight corrected in target lesions was used for further analyses. PET studies were evaluated both visually and semiquantitatively (SUVmax) using a conventional SUVmax cutoff value of 2.5 (significant uptake) which has been considered to provide excellent specificity and sensitivity for detecting lesions [22]. Tumor response was assessed based on RECIST criteria [23]. Patients were considered responders in case of complete (i.e., disappearance of all target lesions) or partial response (i.e., at least a 30% decrease in the sum of the longest diameter of target lesions). Progressive disease was defined as at least 20% increase in the sum of the longest diameter of target lesions or the appearance of one or more new lesions; stable disease was defined as either not sufficient shrinkage to qualify for partial response nor sufficient increase to qualify for progressive disease. Patient's characteristics are reported in Table S1 (Cohort 2). All patients gave their informed written consent to use biological specimens and clinical data for investigational procedures. The study methodologies conformed to the standards set by the Declaration of Helsinki. The study methodologies were approved by the local ethics committee (PROT. N. 20120010288 Titolo: ‘Ruolo del pathway citoprotettivo di TRAP1 e HSP90 nei tumori umani del colon‐retto e della mammella’‐P.I. Prof. M. Lndriscina).

Unless otherwise specified, reagents were purchased from Sigma‐Aldrich (St. Louis, MO, USA). Human CRC HCT116, HT29, CaCo2, and NCIH508 cells were purchased from American Type Culture Collection (ATCC), (Manassas, VA, USA). Cell lines were routinely monitored in our laboratory by microscopic morphology, while cell line authentication was verified before starting this study by STR profiling, according to ATCC product description. TRAP1‐stable interfered HCT116 cells were cultured as previously described [24]. Spheroids derived from human CRCs were kindly provided by Prof. Ruggero De Maria (General Pathology Institute, Catholic University, Rome, Italy). Spheroids were cultured in ultralow attachment tissue culture flasks (Corning Costar, Cambridge, MA, USA) in humidified atmosphere at 37 °C and 5% CO_2_ in a medium containing Advanced DMEM F12 supplemented with 10 mm HEPES, 100 ng·mL^−1^ human recombinant bFGF, 10 mm nicotinamide, 2 mm l‐glutamine, 100 U·mL^−1^ P/S, N2 supplement 1×, B27 supplement 1×, 50 ng·mL^−1^ human recombinant EGF (Thermo Fisher Scientific, Waltham, MA, USA). Spheroids were passaged weekly by mechanical dissociation or by incubation for 3–5 min at 37 °C with TrypLE Express (Thermo Fisher).

### 2.2 Transfection procedures

TRAP1 and BRAF transient silencing were performed with siRNAs purchased from Qiagen (Milano, Italy; cat. No° SI00115150 for TRAP1, cat. No° SI00299488 for BRAF and cat. No° SI00040663 for PFK1). For control experiments, cells were transfected with a similar amount of control siRNA (Qiagen; cat. no. SI03650318). Transient transfections of siRNAs were performed using HiPerFect Transfection Reagent (Qiagen) according to the manufacturer's protocol. Full‐length TRAP1 cDNA, TRAP1 deletion mutant Δ1‐59 TRAP1‐Myc [20, 24], and BRAF V600E (kindly provided by M. Santoro, University of Naples) [25] constructs were cloned in pcDNA3.1 vector (Invitrogen, Carlsbad, CA, USA) and transiently transfected using the PolyFect Transfection Reagent (Qiagen) according to the manufacturer's protocol. TRAP1 stable interference was achieved by transfecting HCT116 cells with TRAP1 (TGCTGTTGACAGTGAGCGACCCGGTCCCTGTACTCAGAAATAGTGAAGCCACAGATGATTTCTGAGTACAGGGACCGGGCTGCCTACTGCCTCGGA) or scrambled (sequence containing no homology to known mammalian genes) shRNA (Open Biosystem, Huntsville, AL, USA) [20].

### 2.3 Immunoblot analysis

Cell pellets and tumor samples were lysed in ice‐cold RIPA buffer as described in Landriscina *et al*. [20]. Subcellular fractions were purified by Qproteome Mitochondria Isolation Kit (Qiagen), according to manufacturer's protocol. Immunoblot analysis was performed as previously reported [20]. Protein immunoprecipitation was carried out starting from 1 mg of total protein extracts by Pierce Classic IP Kit (Thermo Scientific) according to manufacturer's protocol. Protein levels were quantified by densitometric analysis using the quantity one 4.5 software (Bio‐Rad Laboratories GmbH, Hercules, CA, USA) and normalized for the respective housekeeping gene. TRAP1 and GLUT1 expression in tumors was considered upregulated if > 3 times compared to expression levels in normal non‐infiltrated peritumoral mucosa [19]. This cutoff was determined by applying Youden's index [26]. The following antibodies from Santa Cruz Biotechnology (Dallas, TX, USA) were used as follows: mouse monoclonal anti‐HSP75 (TRAP1, sc‐73604), mouse monoclonal anti‐BRAF (sc‐5284), mouse monoclonal anti‐PFK1 (sc‐514824), rabbit polyclonal anti‐MCT4 (sc‐50329), mouse monoclonal anti‐ACTIN (sc‐47778), mouse monoclonal anti‐Tubulin (sc‐8035), rabbit polyclonal anti‐Calnexin (M‐108, sc‐5627), and mouse monoclonal anti‐GAPDH (sc‐47724). The following antibodies were also used as follows: rabbit polyclonal anti‐PFK1 (ab154804; Abcam, UK), rabbit polyclonal anti‐GLUT1 (ab32551; Abcam, Cambridge, UK), and rabbit polyclonal anti‐Hexokinase II (cat. n. 2867S; Cell Signaling, Danvers, MA, USA).

### 2.4 Proximity ligation assay

Cells were seeded on coverslips and, after 48 h, fixed with 4% (w/v) paraformaldehyde in PBS for 20 min. Cells were blocked and permeabilized with 0.4% (w/v) BSA, 0.1% (v/v) Triton X‐100, 5% (v/v) FBS in PBS for 15 min at RT before staining over night with indicated primary antibodies. The Duolink *In Situ* Red Starter Kit Mouse/Rabbit from Sigma‐Aldrich (DUO92101) was used, according to the manufacturer's instructions. Image acquisition was performed by confocal laser‐scanning microscopy using Zeiss 510 LSM from Carl Zeiss Microimaging (Oberkochen, Germany).

### 2.5 Metabolic assays

Glucose uptake was evaluated by both colorimetric and radioactive assays, measuring, respectively, 2‐DG and ^18^F‐FDG uptake. Colorimetric assay was used according to the manufacturer's protocol (ab136955; Abcam). Radioactive assay was previously described by Maddalena *et al*. [27].

Lactate production, phosphofructokinase (PFK) 1 and hexokinase (HK) II activities, and citrate concentration were measured using, respectively, the Lactate Assay Kit (Abcam ab65331), the Sigma‐Aldrich MAK093 and the Abcam ab136957 colorimetric assays and the Citrate Assay Kit (Sigma‐Aldrich MAK057), according to the manufacturer's instructions. Oxygen consumption rates (OCR) in intact cells were measured by Hansatech Oxygraph in a thermostatically controlled chamber (*T* = 37 °C) equipped with a stirring device and a gas‐tight plug with a narrow port enabling addition by microsyringe. Cultured cells were detached, centrifuged at 290 ***g*** for 5 min, and resuspended in standard medium. After stabilization of the oxygraph baseline signal, the cell suspension was added into the reaction chamber at the desired concentration (typically 15–20 × 10^6^ cells·mL^−1^). After achievement of the stationary resting oxygen consumption rate (OCR_R_), 1.0 μg·mL^−1^ of oligomycin was added in order to determine the ATP‐linked respiration (OCRo), followed by the addition of 10 μm of the uncoupler FCCP to obtain the maximal respiratory activity (OCR_U_). The measured OCRs were corrected for the 2 μm rotenone‐insensitive respiration, to be attributable to the mitochondrial respiration, and normalized to the cell number. In specific experiments (Fig. 4), glycolytic metabolism and OXPHOS were measured with Seahorse XF24 Extracellular Flux Analyzer (Agilent, Santa Clara, CA, USA) according to the manufacturer's instructions. Briefly, HCT116 cells were grown in Mc'Coys supplemented with 10% FBS. Twelve hours before the experiment, 1.2 × 10^4^ cells were seeded in a 96‐well microplate, and sodium pyruvate, metabolic drugs, or PBS were injected during measurements.

### 2.6 Cell cycle detection

Cells were plated into 6‐well plates and treated as reported in figure legends, incubated in a culture medium supplemented with 20 μmol·L^−1^ 5‐bromo‐2′‐deoxyuridine (BrdUrd) for 20 min, harvested, washed, and fixed in cold 70% ethanol overnight at 4 °C. After fixation, samples were prepared as reported in Sisinni *et al*. [28]. Finally, samples were analyzed using the FACSCalibur (Becton Dickinson, Franklin Lakes, NJ, USA).

### 2.7 Statistics

The Mann–Whitney test was used to establish the statistical significance between different levels of gene expression in Real‐time PCR analysis, metabolic results, or cells in S‐phase in silenced/transfected/treated cell lines and related controls. The ANOVA and Bonferroni test was used to compare more than two groups. Data are reported as mean value of least three independent experiments (±SD). The Spearman test was used to establish statistical significance of TRAP1, GLUT1 and SUV correlation in human CRCs.

## 3 Results

### 3.1 TRAP1 levels correlate with GLUT1 expression and ^18^F‐FDG uptake in human colorectal carcinomas

**Fig. 1:**
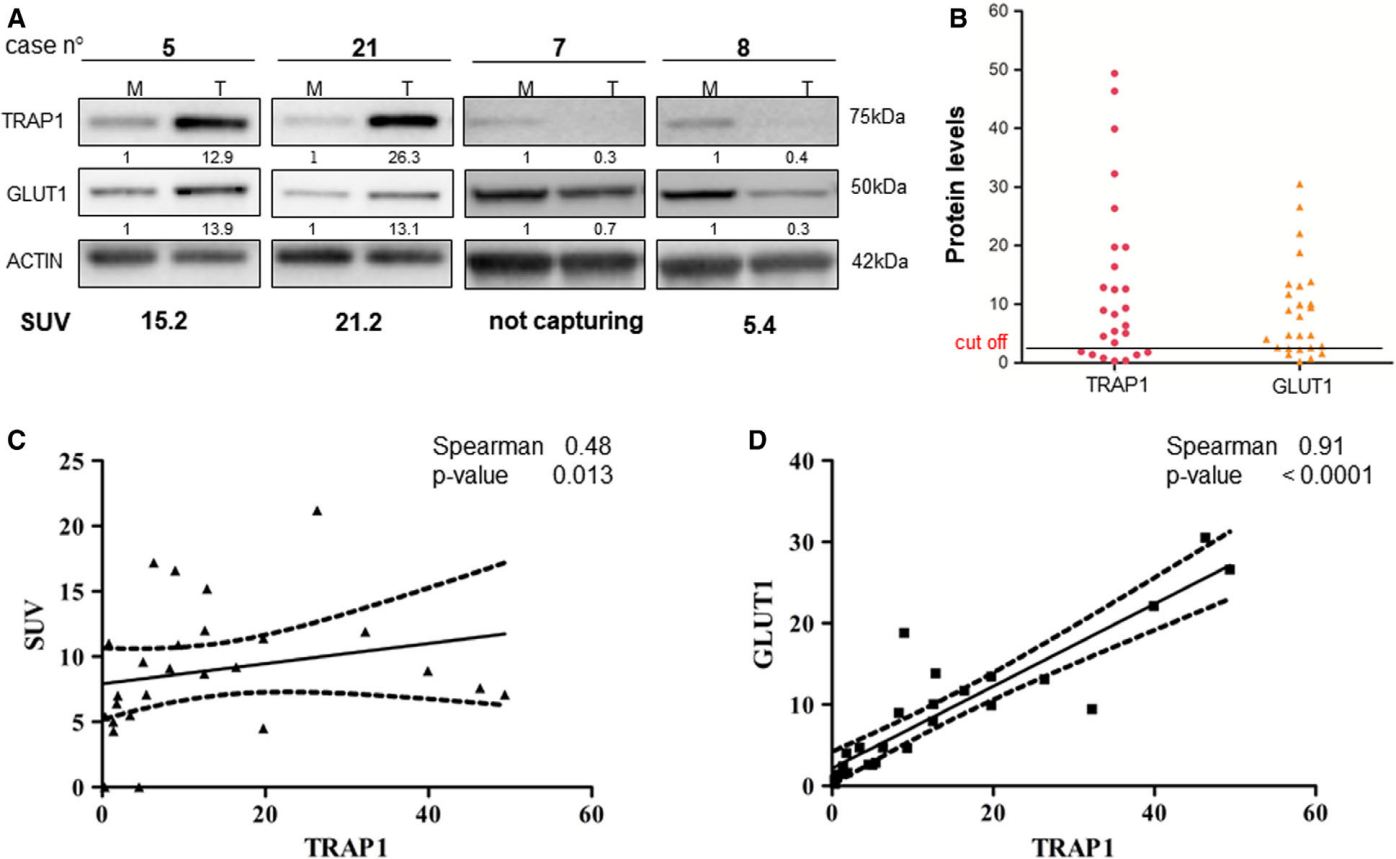
TRAP1 expression correlates with GLUT1 levels and ^18^F‐FDG uptake in human colorectal carcinomas. (A) TRAP1 and GLUT1 immunoblot analysis in four representative cases of human CRCs. (B) TRAP1 and GLUT1 protein levels in the cohort of 26 human CRCs. (C, D) Scatter plots representing the statistical correlation between TRAP1 and SUV upon ^18^F‐FDG PET scan (C) or GLUT1 expression (D) in the cohort of 26 human CRCs.

The expression of TRAP1 and GLUT1 was analyzed in 26 human CRCs enrolled in this study for having been evaluated by ^18^F‐FDG PET scan before surgical intervention (Table S1, Cohort 1). Interestingly, the expression of TRAP1 was confirmed to be upregulated in 73% of primary CRCs, whereas GLUT1 was overexpressed in 65% of cases (Fig. 1A,B and Table S2). The Spearman correlation test showed a week, but statistically significant correlation between TRAP1 protein levels and glucose uptake (measured as standardized uptake value upon PET scan; Fig. 1C) and a more robust correlation between TRAP1 and GLUT1 expression (Fig. 1D). As expected, a correlation was also observed between SUV upon ^18^F‐FDG PET imaging and GLUT1 protein levels (Fig. S1). These results suggest that TRAP1 is involved in the glycolytic phenotype of human CRCs.

### 3.2 TRAP1 regulates the balance between glycolysis and oxidative metabolism in human colorectal carcinoma cell lines

**Fig. 2:**
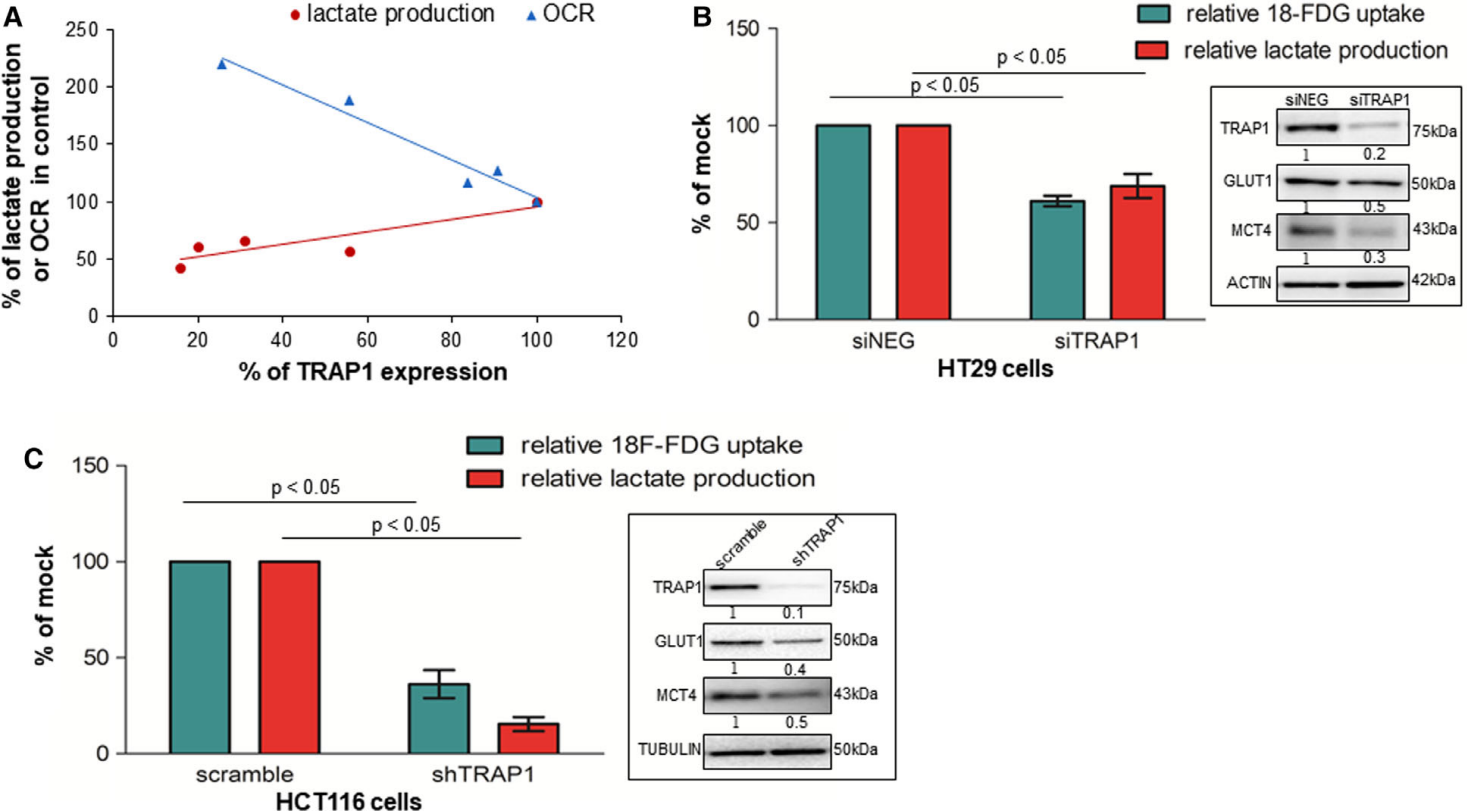
TRAP1 regulates the balance between glycolytic and oxidative metabolism in human CRC cell lines. (A) Plot representing the relationship between TRAP1 expression levels and lactate production or mitochondrial oxygen consumption rate (OCR). Results are plotted from independent siTRAP1 preparations with different efficiency of protein expression and are expressed as percentage of the corresponding Mock control. (B) Relative lactate production and ^18^F‐FDG uptake in transient TRAP1‐silenced HT29 cells. The Mann–Whitney test was used to establish the statistical significance. siNEG vs siTRAP1,^18^F‐FDG uptake: *P* < 0.05, lactate production: *P* < 0.05. (C) Relative lactate production and ^18^F‐FDG uptake in stable TRAP1‐silenced HCT116 cells. The Mann–Whitney test was used to establish the statistical significance scramble vs shTRAP1 ^18^F‐FDG uptake: *P* < 0.05, lactate production: *P* < 0.05. (B, C) Inserts: TRAP1, GLUT1, and MCT4 immunoblot analysis in, respectively, TRAP1‐silenced HT29 (B) and HCT116 (C) cells.

TRAP1 role in metabolism was further explored in CRC cell lines transiently silenced for TRAP1 expression to evaluate lactate production and oxygen consumption rate (OCR). Results from independent siTRAP1 preparations with different efficiency of protein expression, reported as percentage of the corresponding mock controls, suggest a linear correlation between TRAP1 protein expression and lactate production in HCT116 cells (Fig. 2A) and an inverse correlation between TRAP1 protein levels and mitochondrial respiratory activity with OCR progressively increased upon TRAP1 downregulation (Fig. 2A). Row data of these experiments are reported in Fig. S2A. The dependency of lactate production from TRAP1 expression was confirmed upon transient or stable TRAP1 knock down in, respectively, HT29 (Fig. 2B) and HCT116 (Fig. 2C and Fig. S2B). Consistently, glucose uptake was significantly decreased by 40–60% after transient/stable TRAP1 silencing in HT29 (Fig. 2B) and HCT116 (Fig. 2C and Fig. S2B). Finally, a significant reduction in the expression of the plasma membrane glucose transporter, GLUT1 and the lactate transporter, MCT4 was observed in HCT116 and HT29 cells upon TRAP1 silencing (Fig. 2B,C and Fig. S2B, inserts). Since HCT116 and HT29 cells are routinely cultured in the presence of high glucose (16.6 mm), to rule out the hypothesis that the glycolytic profile of CRC cell lines with high TRAP1 expression may depend on glucose concentration, metabolic parameters were measured in HCT116 cells cultured in more physiologic glucose concentration (5.5 mm) upon TRAP1 silencing. As reported in Fig. S2C,D, TRAP1 effect on glycolytic metabolism and OXPHOS is preserved, although attenuated, even in the presence of low glucose. Indeed, glucose uptake and lactate production are significantly reduced upon TRAP1 silencing (Fig. S2C) and this is paralleled by a significant increase in OCR (Fig. S2D). Altogether, these data suggest that high TRAP1 expression favors glycolytic metabolism in human CRC cells.

### 3.3 TRAP1 glycolytic phenotype is independent from its quality control on BRAF

Since TRAP1 is responsible for quality control of BRAF protein synthesis [25] and BRAF signaling enhances Warburg metabolism [29, 30], the hypothesis that TRAP1 regulation of glucose metabolism may occur through the modulation of BRAF signaling was further evaluated. To this purpose, glucose uptake and lactate production were evaluated in BRAF V600E HT29 cells upon BRAF silencing and parallel TRAP1 upregulation (Fig. S3A). Interestingly, while BRAF downregulation induced a statistically significant inhibition of both glucose uptake and lactate production, TRAP1 overexpression completely rescued the effect of BRAF silencing on glucose metabolism (Fig. S3A). In parallel experiments, BRAF V600E mutant was upregulated in TRAP1‐silenced HCT116 cells and cell lines evaluated for glucose uptake and lactate production (Fig. S3B). Indeed, BRAF upregulation resulted in a marked increase of 2‐DG uptake and a more modest increase in lactate production. However, the downregulation of both glucose uptake and lactate production in a low TRAP1 background was conserved independently from BRAF V600E mutant upregulation (Fig. S3B). These results suggest that TRAP1 regulation of glucose metabolism is independent from its quality control on BRAF synthesis.

### 3.4 TRAP1 enhances glycolysis through regulation of PFK1 activity/expression

**Fig. 3:**
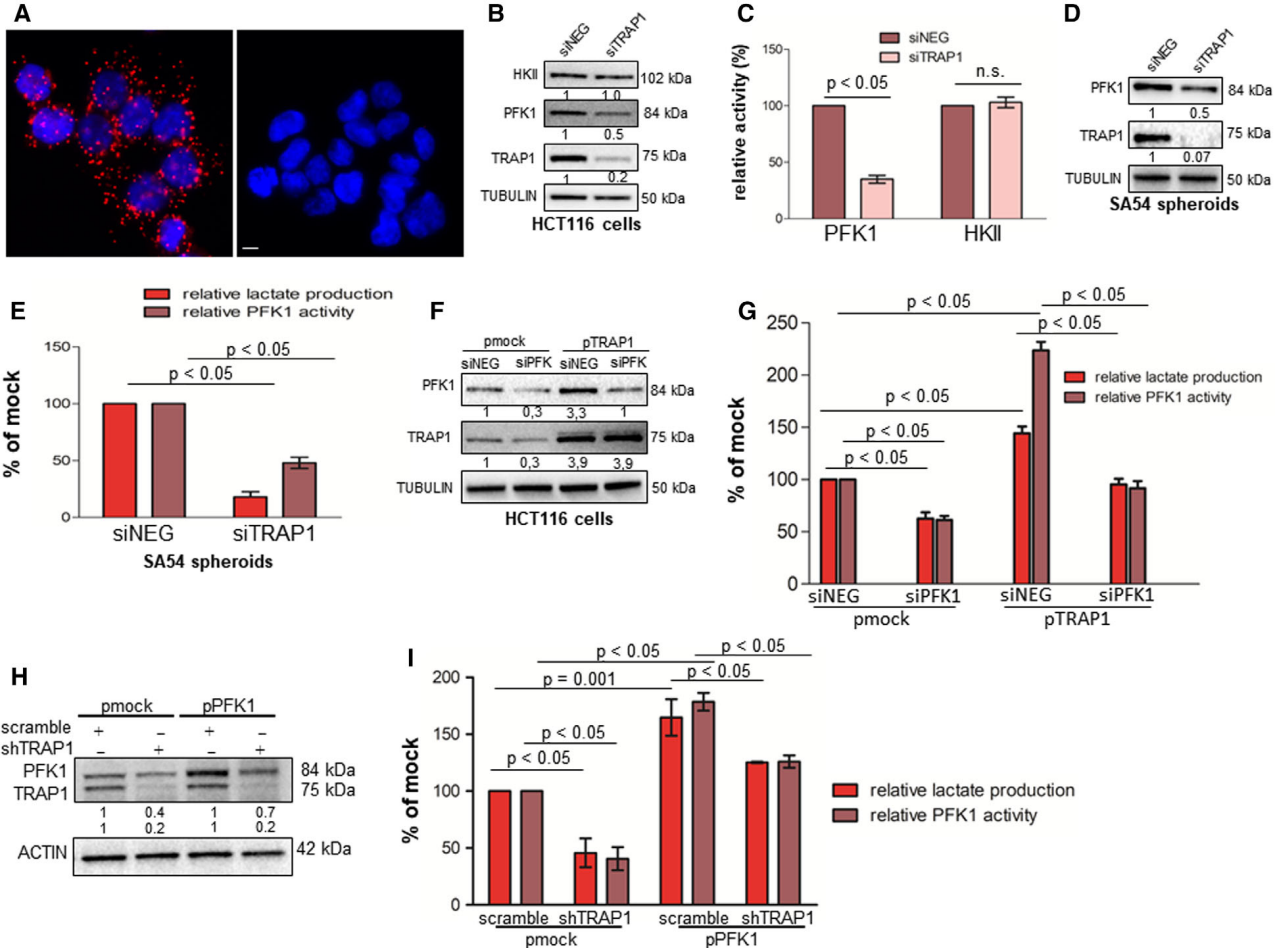
TRAP1 regulates Warburg metabolism through modulation of PFK1 activity/expression. (A) Representative fluorescence images showing proximity ligation assay signals (red), detected in HCT116 cells stained with TRAP1 and PFK1 (left panel). The right panel represents the negative control stained with not related antibodies. Nuclei are DAPI‐labeled (blue). Scale bar, 10 µm. (B, C) HKII, PFK1, and TRAP1 immunoblot analysis (B) and relative PFK1 and HKII activity (C) in transient TRAP1‐silenced HCT116 cells. (D, E) PFK1 and TRAP1 immunoblot analysis (D), and relative PFK1 activity and lactate production (E) in transient TRAP1‐silenced SA54 CRC spheres. (F, G) PFK1 and TRAP1 immunoblot analysis (F), and relative lactate production and PFK1 activity (G) in PKF1‐silenced HCT116 cells transfected with TRAP1 cDNA. (H, I) PFK1 and TRAP1 immunoblot analysis (H) and relative lactate production and PFK1 activity (I) in shTRAP1 HCT116 cells transfected with PFK1 cDNA. Graphs represent mean ± SD of three experiments. The Mann–Whitney test was used to establish the statistical significance between two groups (*P* < 0.05). (G) ANOVA test: *P* = 0.0001; Bonferroni *post hoc* test: siNEG pmock vs siPFK1, lactate production: *P* < 0.01, PFK1 activity: *P* < 0.01, siNEG pmock vs siNEG pTRAP1, lactate production: *P* < 0.001, PFK1 activity *P* < 0.001; siNEG pTRAP1 vs siPFK1 pTRAP1, lactate production *P* < 0.05, PFK1 activity: *P* < 0.001. (I) ANOVA test: *P* = 0.004; Bonferroni *post hoc* test: scramble pmock vs shTRAP1 pmock, lactate production: *P* < 0.01, PFK1 activity: *P* < 0.01; scramble pPFK1 vs shTRAP1 pPFK1 lactate production: *P* < 0.05, PFK1 activity: *P* < 0.01.

To dissect the molecular mechanism underlying TRAP1 regulation of glycolytic metabolism, we took advantage from our list of putative TRAP1 interactors, obtained by proteomic analysis of TRAP1 immunoprecipitates [20], which, among others, identified phosphofructokinase‐1 (PFK1), a key glycolytic enzyme [31]. Noteworthy, the direct protein–protein interaction between TRAP1 and PFK1 was confirmed in HCT116 cells by *in situ* proximity ligation assay (Fig. 3A). Since TRAP1 is a molecular chaperone responsible for cotranslational quality control on nascent polypeptides [32], PFK1 expression and activity were evaluated upon TRAP1 silencing in HCT116 cells cultured in standard medium or in the presence of low glucose. Indeed, PFK1 protein level (Fig. 3B and Fig. S4A) and activity (Fig. 3C) were conserved in a high TRAP1 background, but significantly reduced upon TRAP1 transient silencing (Fig. 3B,C). Similar data were observed in HCT116 cells cultured in low glucose (Fig. S4B). Hexokinase II (HKII) protein level and activity were unchanged in the same experimental conditions (Fig. 3B,C). Of note, no significant modification of PFK1 mRNA expression was observed under TRAP1 silencing conditions (Fig. S4C).

Based on the rationale that colon cancer stem cells (CSCs) are characterized by a predominant glycolytic metabolism [33, 34] and that TRAP1 is upregulated in colon CSCs and involved in their maintenance [11], patient‐derived CRC spheres, known to be enriched in CSCs [35], were evaluated to further establish the relationship between TRAP1, PFK1 activity/expression, and lactate production. Interestingly, TRAP1 silencing resulted in parallel downregulation of PFK1 protein levels (Fig. 3D) and activity and lactate production (Fig. 3E) in patient‐derived SA54 CRC spheres, this confirming analogous data shown in Fig. 3B,C.

In order to evaluate whether TRAP1 regulation of Warburg metabolism depends on its effect on PFK1 activity, PFK1 was knocked down in TRAP1‐overexpressing HCT116 cells. Of note, while TRAP1 upregulation resulted in enhanced PKF1 expression/activity and lactate production, PFK1 protein/activity downregulation in a high‐TRAP1 background significantly inhibited lactate production (Fig. 3F,G). Consistently, PFK1 expression/activity was reduced in stable TRAP1 silenced HCT116 cells and TRAP1 re‐expression in a low TRAP1 background resulted in enhanced PFK1 expression/activity and lactate production (Fig. S4D). Finally, PFK1 was re‐expressed in shTRAP1 HCT116 cells, thus resulting in a partial, but statistically significant re‐establishment of PFK1 activity and lactate production (Fig. 3H,I). Altogether, these data suggest that TRAP1 modulates glycolytic metabolism through the regulation of PFK1 activity/expression.

### 3.5 TRAP1 regulation of glycolytic metabolism and PFK1 activity depends on mitochondrial respiratory capacity

**Fig. 4:**
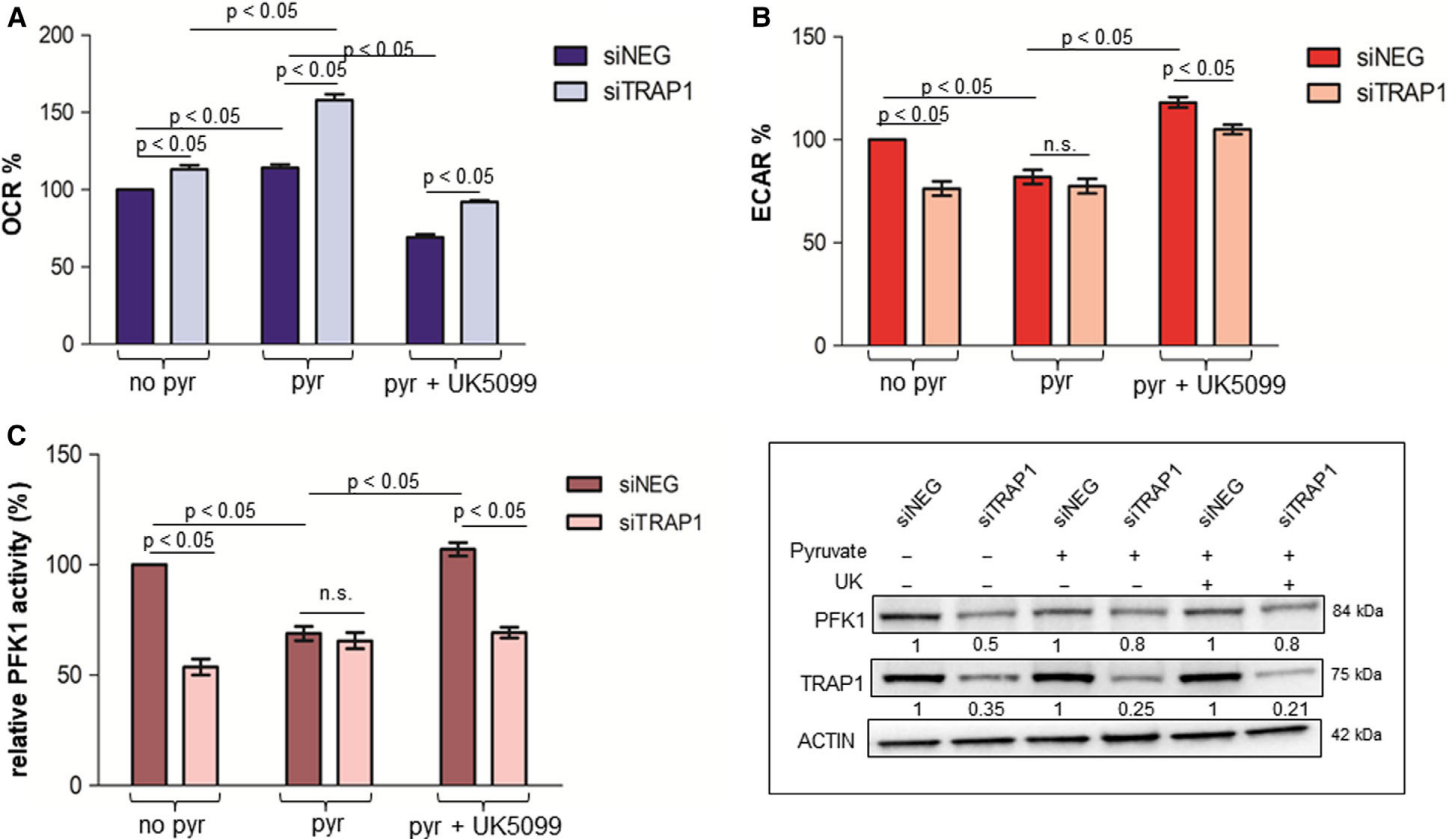
TRAP1 regulation of PFK1 and glycolytic metabolism depends on mitochondrial respiratory capacity. (A–C) Seahorse analysis of OCR (A) and ECAR (B) and PFK1 activity (C) in TRAP1‐silenced HCT116 cells cultured in standard medium (no pyr), supplemented with 10 mm pyruvate for 6 h (pyr) or incubated with 5 μm UK5099 (pyr+UK5099) for 6 h. Insert: PFK1 and TRAP1 immunoblot analysis in TRAP1‐silenced HCT116 cells cultured in standard medium, supplemented with 10 mm pyruvate for 6 h or incubated with 5 μm UK5099 for 6 h. Graphs represent mean ± SD of three experiments. The Mann–Whitney test was used to establish the statistical significance between two groups (*P* < 0.05). (A) ANOVA test: *P* = 0.0005; Bonferroni *post hoc* test: no pyr: *P* < 0.05, pyr: *P* > 0.001; pyr+UK5099: *P* < 0.001. (B) ANOVA test: *P* = 0.003; Bonferroni *post hoc* test: no pyr: *P* < 0.01; pyr: *P* > 0.05; pyr+UK5099: *P* < 0.05. (C) ANOVA test: *P* = 0.0027; Bonferroni *post hoc* test: no pyr: *P* < 0.001; pyr: *P* > 0.05; pyr + UK5099: *P* < 0.001.

In order to evaluate the role of TRAP1 in regulating the balance between glycolytic and oxidative metabolism in CRC cell lines, we generated a model of cell supplementation with pyruvate to boost OXPHOS. Cell metabolism was analyzed by Seahorse technology to evaluate in parallel mitochondrial OCR (Fig. 4A) and extracellular acidification rate (ECAR; Fig. 4B) upon TRAP1 silencing; PFK1 activity was evaluated in the same experimental conditions (Fig. 4C). As previously observed, high TRAP1 expression correlated with lower OCR (Mann–Whitney test: *P* < 0.05; Bonferroni *post hoc* test: *P* < 0.05) and higher ECAR (Mann–Whitney test: *P* < 0.05; Bonferroni *post hoc* test: *P* < 0.01) and PFK1 activity (Mann–Whitney test: *P* < 0.05; Bonferroni *post hoc* test: *P* < 0.001) in standard conditions (lack of pyruvate supplementation). As expected, pyruvate supplementation enhanced OCR independently from TRAP1 expression with maximal induction in conditions of TRAP1 silencing (Mann–Whitney test: *P* < 0.05) and this was paralleled by downregulation of ECAR (Mann–Whitney test: *P* < 0.05) and PFK1 activity (Mann–Whitney test: *P* < 0.05) to levels observed in conditions of low TRAP1 background. Since these data suggest that TRAP1 regulation of PFK1 activity occurs to balance the suppression of OXPHOS, TRAP1‐silenced cells were treated with UK5099, a pharmacological inhibitor of pyruvate transport in mitochondria. Interestingly, inhibition of pyruvate uptake into mitochondria resulted in a significant inhibition of OCR (Mann–Whitney test: *P* < 0.05; Bonferroni *post hoc* test: *P* < 0.001) and re‐establishment of TRAP1 control of ECAR (Mann–Whitney test: *P* < 0.05; Bonferroni *post hoc* test: *P* < 0.05) and PFK1 activity (Mann–Whitney test: *P* < 0.05; Bonferroni *post hoc* test: *P* < 0.001). These data suggest that TRAP1 regulation of lactate production and PFK1 activity is maximized in conditions of low OXPHOS.

**Fig. 5:**
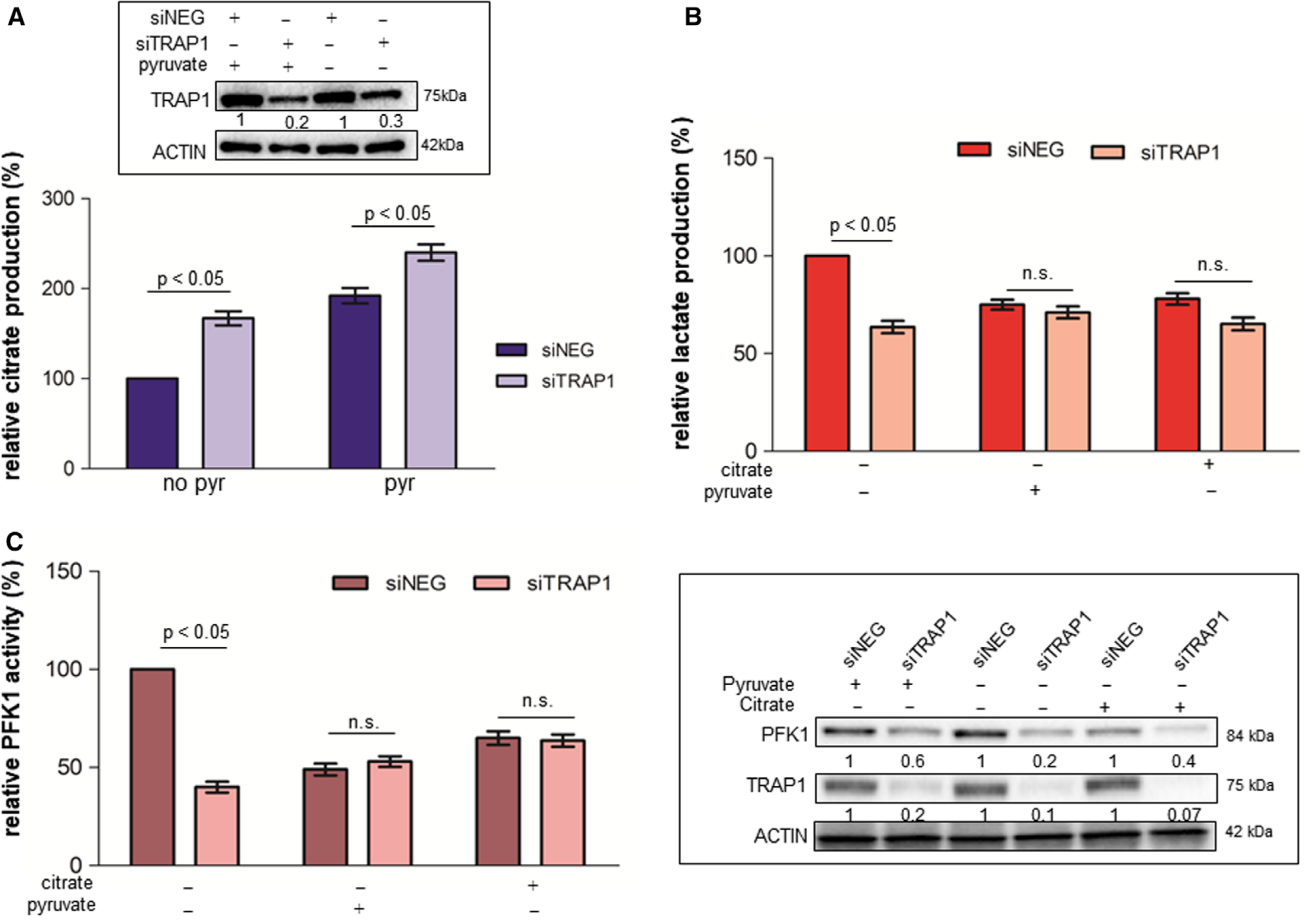
TRAP1 regulation of PFK1 and glycolytic metabolism depends on citrate production. (A) Citrate concentration in TRAP1‐silenced HCT116 cells cultured in standard medium (no pyruvate) or supplemented with 10 mm pyruvate for 6 h. (B, C) Relative lactate production (B) and PFK1 activity (C) in TRAP1‐silenced HCT116 cells cultured in standard medium (no pyruvate), or supplemented with 10 mm pyruvate or 1 mm citrate for 6 h. Insert: PFK1 and TRAP1 immunoblot analysis in TRAP1‐silenced HCT116 cells cultured in standard medium (no pyruvate) or supplemented with 10 mm pyruvate or with 1 mm citrate for 6 h. Graphs represent mean ± SD of three experiments. The Mann–Whitney test was used to establish the statistical significance between two groups (*P* < 0.05). (A) ANOVA test: *P* = 0.0005; Bonferroni *post hoc* test: no pyr: *P* < 0.05, pyr: *P* > 0.001; pyr+UK5099: *P* < 0.001. (B) ANOVA test: *P* = 0.003; Bonferroni *post hoc* test: no pyr: *P* < 0.01; pyr: *P* > 0.05; pyr+UK5099: *P* < 0.05. (C) ANOVA test: *P* = 0.0027; Bonferroni *post hoc* test: no pyr: *P* < 0.001; pyr: *P* > 0.05; pyr+UK5099: *P* < 0.001.

Citrate is an intermediate product of tricarboxylic acid (TCA) and an allosteric inhibitor of PFK1, known to slow glycolysis, enhance OXPHOS, and link mitochondrial TCA cycle with rate of glycolysis [36, 37]. Thus, citrate levels were evaluated in HCT116 cells supplemented with pyruvate and/or silenced for TRAP1. Interestingly, citrate levels were confirmed to be increased in HCT116 cells incubated in the presence of pyruvate and silenced for TRAP1, both conditions of enhanced mitochondrial respiration (Fig. 5A). Thus, to test the role of citrate in TRAP1 regulation of the balance between glycolysis and OXPHOS, TRAP1‐silenced HCT116 cells were supplemented with citrate and evaluated for lactate production and PFK1 activity in comparison with cell cultures supplemented with pyruvate. Noteworthy, both lactate production (Fig. 5B) and PFK1 activity (Fig. 5C) were maximal in TRAP1‐expressing cells cultured in the absence of pyruvate; by contrast, citrate supplementation, as observed for pyruvate supplementation, resulted in the loss of TRAP1 capacity to enhance lactate production and PFK1 activity. Altogether, these data suggest that TRAP1 regulates Warburg metabolism to balance modification of OXPHOS and that this process occurs through intermediates of the TCA cycle and the modulation of PFK1 activity/expression.

### 3.6 TRAP1 regulation of PFK1 glycolytic activity depends on their interaction on the endoplasmic reticulum

**Fig. 6:**
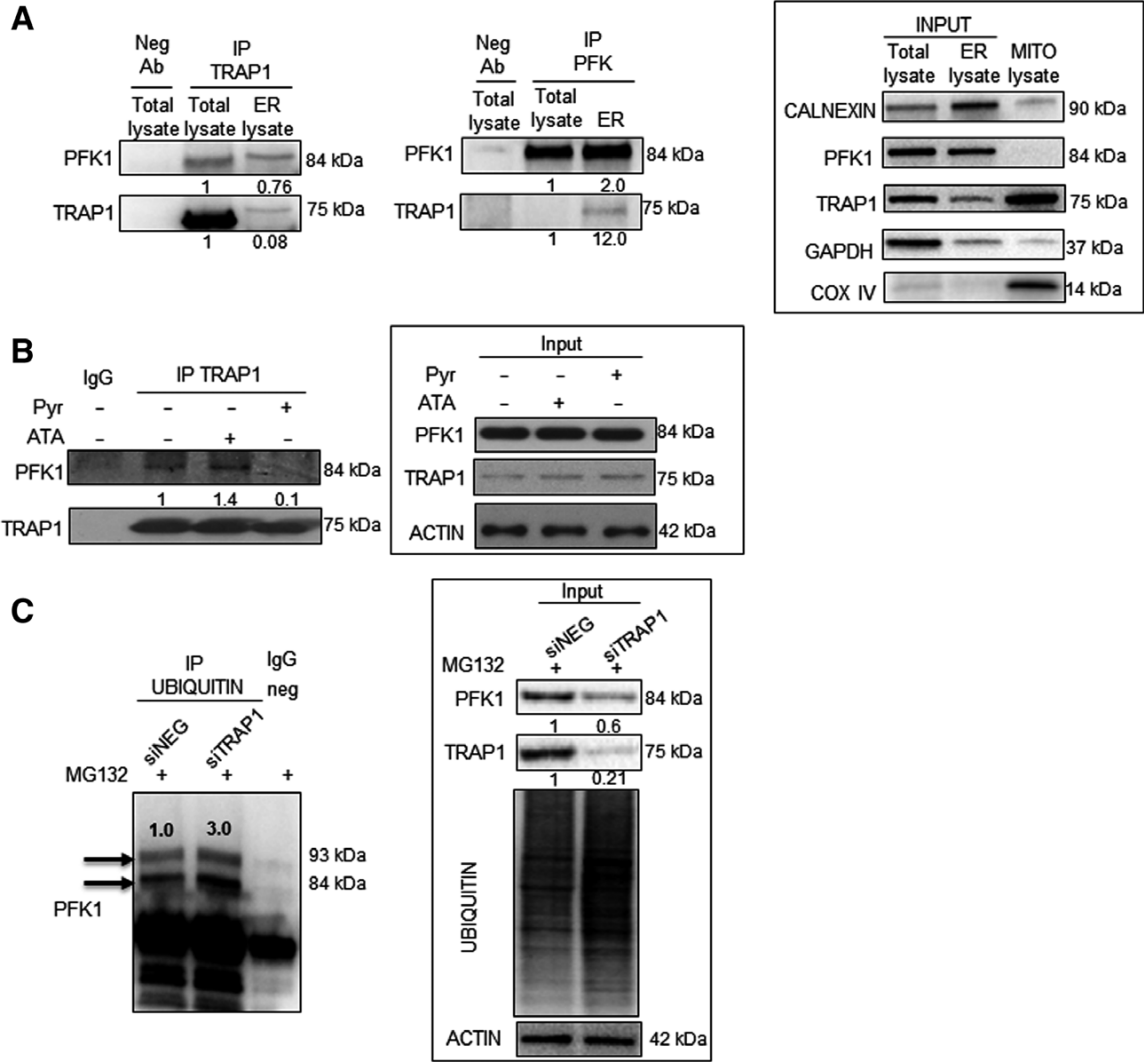
TRAP1 interacts with PFK1 and regulates its activity/stability. (A) PFK1 (left panel) and TRAP1 (right panel) immunoblot analysis of immunoprecipitates with anti‐TRAP1 (left panel) or anti‐PFK1 (right panel) antibodies from total lysates and ER fractions of HCT116 cells. Neg Ab, total cellular extracts incubated with not related antibody; IP, immunoprecipitation with the corresponding antibodies. Input: PFK1 and TRAP1 immunoblot analysis of total lysates and ER fractions of HCT116 cells. (B) PFK1 immunoblot analysis of immunoprecipitates with anti‐TRAP1 antibodies from total lysates of HCT116 cells cultured in standard medium or supplemented with 10 mm pyruvate for 6 h (pyr) or 50 μm antimycin A (ATA) for 1 h. Insert: PFK1 and TRAP1 immunoblot analysis in HCT116 cells cultured in standard medium or supplemented with 10 mm pyruvate for 6 h (pyr) or 50 μm antimycin A (ATA) for 1 h. (C) PFK1 immunoblot analysis of immunoprecipitates with anti‐ubiquitin antibodies from total lysates of TRAP1‐silenced HCT116 cells cultured in the presence of 10 μm MG132 for 2 h. Insert: PFK1, TRAP1, and anti‐ubiquitin immunoblot analysis in TRAP1‐silenced HCT116 cells.

TRAP1 interacts with its client proteins (i.e., sorcin, BRAF, CDK1, F1ATPase) and is responsible for their quality control through the cooperation with the proteasome regulatory particle, TBP7 [20, 24, 25, 28]. Thus, we hypothesized that PKF1 is among TRAP1 client proteins whose activity is regulated upon their reciprocal interaction. Thus, the interaction between TRAP1 and PFK1 was investigated by co‐immunoprecipitation (co‐IP) experiments in total lysate and endoplasmic reticulum (ER) fraction of HCT116 cells. Indeed, PFK1 immunoblot analysis of TRAP1 co‐immunoprecipitates detected an 84 kDa band in both total lysate and the ER fraction (Fig. 6A, left panel). Consistently, TRAP1 immunoblot analysis of PFK1 co‐immunoprecipitates detected a 75 kDa band only in the ER fraction (Fig. 6A, right panel). Since PFK1 activity is downregulated upon pyruvate supplementation, PFK1 co‐immunoprecipitation was evaluated in the presence and absence of pyruvate to address the hypothesis that TRAP1/PFK1 interaction is relevant for PKF1 activity regulation. PFK1 co‐immunoprecipitation was evaluated also upon incubation with antimycin A, an inhibitor of cellular respiration, as a control. Noteworthy, pyruvate supplementation, but not OXPHOS inhibition, resulted in loss of PFK1/TRAP1 interaction, being PFK1 detected in TRAP1 co‐immunoprecipitates only in cells cultured in standard conditions (lack of pyruvate) or exposed to antimycin A (Fig. 6B). In addition, since TRAP1 silencing results in downregulation of PFK1 protein level/activity, we hypothesized that the downregulation of PFK1 may be dependent on increased ubiquitination/degradation of the inactive protein. Thus, ubiquitinated proteins were immunoprecipitated from TRAP1‐silenced HCT116 cells and further evaluated by anti‐PFK1 antibodies to assess the level of ubiquitinated PFK1. Noteworthy, PFK1 immunoblot analysis of anti‐ubiquitin immunoprecipitates showed increased levels of PFK1 polyubiquitination in TRAP1‐interfered cells despite the overall reduction in PFK1 levels in total lysates (Fig. 6C) and this is consistent with increase in overall protein ubiquitination in TRAP1‐silenced cells (Fig. 6C, insert). Finally, since TRAP1 is mostly localized in mitochondria (Fig. S5A), in order to further characterize the role ER‐associated TRAP1 in regulating Warburg metabolism, shTRAP1 HCT116 cells were transfected with a TRAP1 deletion mutant lacking the mitochondrial targeting sequence and unable to accumulate in mitochondria (D1‐59 TRAP1‐Myc; Fig. S5B) [24] and evaluated for PFK1 expression/activity and lactate production. Noteworthy, the upregulation of the ER‐associated TRAP1 deletion mutant in a low TRAP1 background rescued PFK1 expression/activity and lactate production (Fig. S5C). These data suggest that TRAP1 and PFK1 interact on the ER and that this interaction is relevant to maximize PFK1 glycolytic activity, being the protein ubiquitinated and degraded in a low TRAP1 background.

### 3.7 Inhibition of TRAP1‐dependent glycolytic phenotype potentiates cell response to EGFR inhibition

**Fig. 7:**
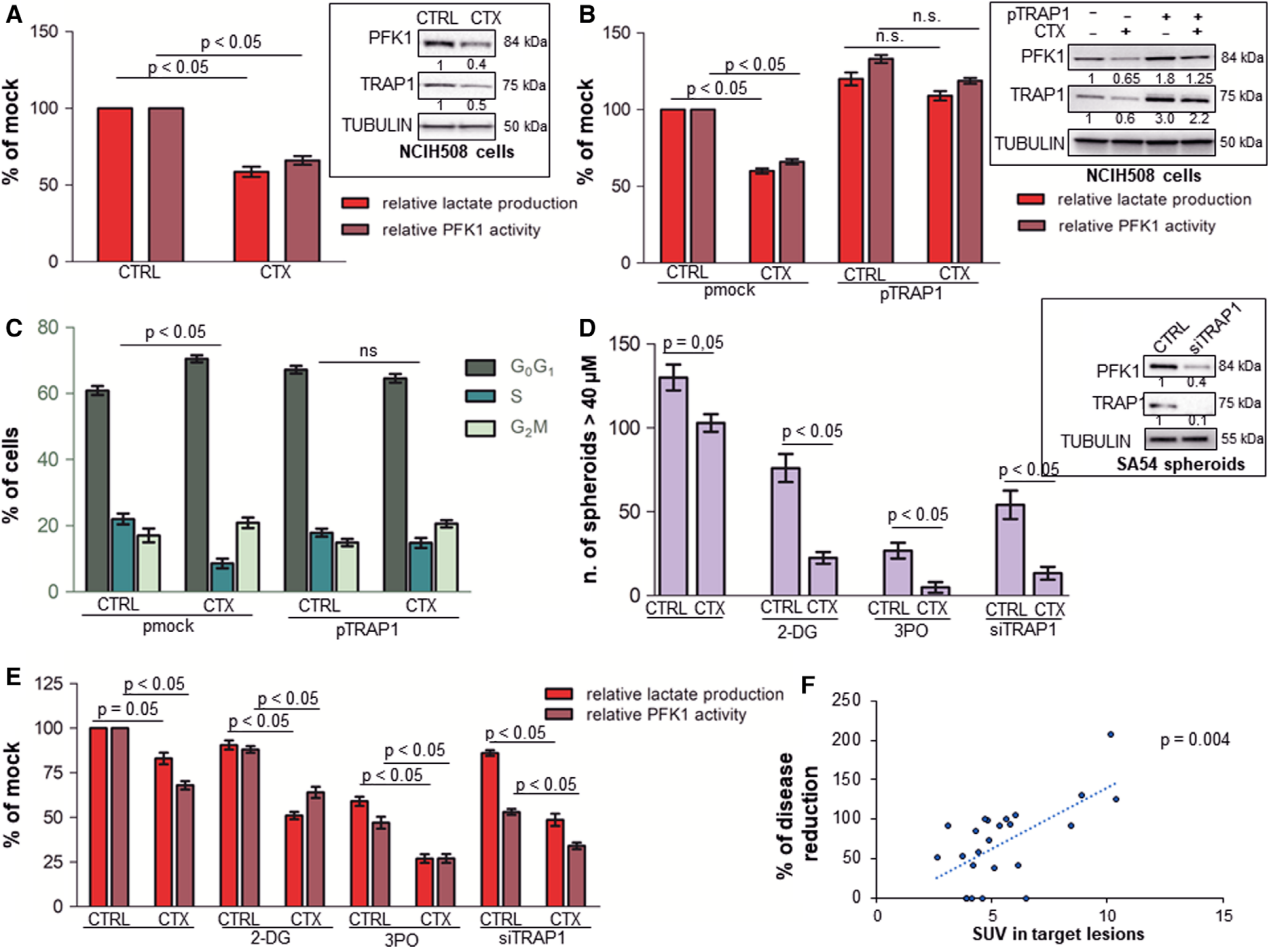
High TRAP1 expression and glycolytic metabolism correlate with poor response to cetuximab *in vitro* and *in vivo*. A. Relative lactate production and PFK1 activity in RAS‐wild‐type NCIH508 cells incubated with 17 nm cetuximab for 15 h. Insert: PFK1 and TRAP1 immunoblot analysis in NCIH508 cells incubated with 17 nm cetuximab for 15 h. (B, C) Relative lactate production and PFK1 activity (B) and cell cycle distribution (C) in NCIH508 cells transfected with TRAP1 cDNA and incubated with 17 nm cetuximab for 15 h. (B) Insert: PFK1 and TRAP1 immunoblot analysis in NCIH508 cells transfected with TRAP1 cDNA and incubated with 17 nm cetuximab for 15 h. (D, E) Number of spheroid (D), relative lactate production and PFK1 activity (E) in RAS‐wild‐type patient‐derived CRC spheroids (SA54 cells) incubated with 680 nm cetuximab, 10 μm 2DG, 10 μm 3PO for 48 h or silenced for TRAP1 or exposed to the combination of cetuximab and 2DG, 3PO, or TRAP1 silencing. Spheroids were counted if they were ≥ 40 μm in size. (D) Insert: TRAP1 and PFK1 immunoblot analyses in patient‐derived spheroids silenced for TRAP1. (F) Plot representing the relationship between ^18F^FDG PET uptake at baseline and percent of size variation of target lesions according to RECIST criteria in mCRCs treated with first‐line chemotherapy combined with cetuximab. Graphs represent mean ± SD of three experiments. The Mann–Whitney test was used to establish the statistical significance between two groups (*P* < 0.05). B. ANOVA test: *P* = 0.002; Bonferroni *post hoc* test: CTRL pmock vs CTX pmock, lactate production: *P* < 0.001, PFK1 activity: *P* < 0.001; CTRL pTRAP1 vs CTX pTRAP1, lactate production: *P* > 0.05, PFK1 activity: *P* > 0.05; CTRL pmock vs CTRL pTRAP1, lactate production: *P* > 0.001, PFK1 activity: *P* < 0.001. D. ANOVA test: *P* = 0.01; Bonferroni *post hoc* test: CTRL vs CTX *P* > 0.05; CTRL 2‐DG vs CTX 2‐DG *P* < 0.001; CTRL 3PO vs CTX 3PO *P* < 0.001; siTRAP1 vs siTRAP1 CTX *P* < 0.001. E. ANOVA test: *P* < 0.007; Bonferroni *post hoc* test: CTRL vs CTX lactate production: *P* = 0.05, PFK1 activity: *P* < 0.001; CTRL 2‐DG vs CTX 2‐DG lactate production: *P* < 0.001, PFK1 activity *P* < 0.001; CTRL 3PO vs CTX 3PO lactate production: *P* < 0.001, PFK1 activity: *P* < 0.001; siTRAP1 vs siTRAP1 CTX lactate production: *P* < 0.01, PFK1 activity: *P* < 0.001.

Several observations suggest that high glycolytic metabolism favors resistance to EGFR inhibitors in lung carcinoma [38]. Therefore, the relationship between TRAP1 expression, glycolytic metabolism, and response to cetuximab was investigated in RAS‐wild‐type CRC cells and CRC spheres. In preliminary experiments, PFK1 expression/activity and glycolytic metabolism were evaluated in RAS‐wild‐type NCIH508 (Fig. 7A) and CaCo2 (Fig. S6) cells upon exposure to cetuximab. Interestingly, EGFR inhibition resulted in downregulation of TRAP1, PFK1 expression/activity, and lactate production in NCIH508 cells (Fig. 7A) and TRAP1, PFK1, and MCT4 expression in CaCo2 cells (Fig. S6). Thus, TRAP1 was upregulated in NCIH508 cells and cells were evaluated for Warburg metabolism (Fig. 7B) and cell cycle progression (Fig. 7C) in response to cetuximab. Noteworthy, while TRAP1 upregulation resulted in enhancement of PFK1 protein expression/activity and lactate production (Fig. 7B), cetuximab failed to significantly downregulate PFK1 expression/activity and lactate production (Fig. 7B) and cell cycle progression (Fig. 7C) in NCIH508 cells with high TRAP1 background. In parallel experiments, cetuximab activity was evaluated by a spheroid‐formation assay (Fig. 7D and Fig. S7) and for its capacity to inhibit lactate production and PFK1 activity in RAS‐wild‐type CRC spheres (Fig. 7E). Noteworthy, cetuximab showed poor inhibitory activity as single agent (Fig. 7D and Fig. S7) and the inhibition of Warburg metabolism by 2DG, an inhibitor of glycolysis, 3PO, an inhibitor of PFK1, or TRAP1 silencing resulted in attenuation of spheroid formation (Fig. 7D and Fig. S7) and a parallel inhibition of lactate production and PFK1 activity (Fig. 7E). Noteworthy, the combination of cetuximab with 2DG, 3PO, or TRAP1 interference significantly inhibited colony formation (Fig. 7D and Fig. S7) as well as lactate production and PFK1 activity (Fig. 7E). Consistently, cell cycle progression (Fig. S8A), lactate production, and PFK1 activity (Fig. S8B) were evaluated in RAS‐wild‐type Caco2 cells in the same experimental conditions. Indeed, CaCo2 cells showed a minimal/moderate inhibition of cell cycle progression (Fig. S8A), lactate production, and PFK1 activity (Fig. S8B) in response to cetuximab, 2DG, 3PO, or upon TRAP1 silencing. Noteworthy, the combination of cetuximab with 2DG, 3PO, or TRAP1 interference resulted in a more profound inhibition of cell cycle progression (Fig. S8A), lactate production, and PFK1 activity (Fig. S8B). These data suggest that the pharmacological inhibition of EGFR is partially impaired by high Warburg metabolism in RAS‐wild‐type CRC cells and spheres. Consequently, we addressed the hypothesis that high glycolytic metabolism may result in poor cetuximab activity in human metastatic CRCs (mCRCs). To this aim, we analyzed a cohort of 15 human RAS‐wild‐type mCRCs treated with first‐line chemotherapy combined with cetuximab and evaluated by ^18^F‐FDG PET scan before therapy and CT scan after therapy (Table S1, cohort 2). Response to therapy was evaluated by RECIST criteria upon TC imaging and correlated with ^18^F‐FDG uptake at baseline. Noteworthy, ^18^F‐FDG uptake was significantly higher in progressing compared to responding (SUV, 9.8 ± 0.8 vs 4.5 ± 1.1; *P* < 0.0001) or stable tumors (SUV, 9.8 ± 0.8 vs 5.3 ± 1.5; *P* = 0.0007) and a direct correlation was observed between ^18^F‐FDG SUV and lack of response to cetuximab (Spearman correlation test, *R* = 0.555908, *P* = 0.004; Fig. 7F). Altogether, these data provide the proof of concept that metabolic remodeling is a mechanism to escape from EGFR inhibition in RAS‐wild‐type mCRCs and that TRAP1 targeting may represent a strategy to enhance cell response to cetuximab.

## 4 Discussion

Molecular chaperones are involved in cancer cell adaptation to stress conditions, that is, unfavorable environments, hypoxia, ER stress, metabolic stress, drug therapy, and their upregulation is widely considered a mechanism of cancer progression [9, 19, 39, 40, 41, 42, 43, 44]. Furthermore, metabolic rewiring is used by cancer cells to adapt to environmental stresses and molecular chaperones are hub proteins connecting different metabolic pathways and favoring cancer cell reprogramming [5, 45]. We previously demonstrated that TRAP1 protein network is upregulated in human CRCs [18, 19] and its high expression correlates with poor clinical outcome [18], drug resistance [19], and suppression of mitochondrial respiration [15]. Herein, we tested the hypothesis that TRAP1 favors metabolic adaptation in human CRC. Our data suggest that (a) high TRAP1 expression correlates with enhancement of Warburg metabolism in human CRC samples, patient‐derived spheroids, and cell lines; (b) TRAP1 control of glycolysis depends on its interaction with PFK1 on endoplasmic reticulum with regulation of PFK1 activity/stability; and (c) TRAP1 modulates glycolysis and PFK1 activity to balance modifications of mitochondrial respiration. Clinically relevant is the observation that cetuximab activity is partially impaired in high TRAP1 background and increased Warburg metabolism and that high PET ^18^F‐FDG uptake at baseline results in lower cetuximab activity in mCRC first‐line therapy.

These observations shed light on TRAP1 regulation of cancer metabolism and its involvement in CRC biology. Intriguingly, TRAP1 is a key regulator of the balance between oxidative and glycolytic metabolism and high TRAP1 expression favors Warburg metabolism. Indeed, TRAP1 suppresses mitochondrial respiration upon interaction/inhibition of complexes II and IV of the respiratory chain [15, 16] and concomitantly enhances GLUT1 expression, glucose uptake, PFK1 activity/stability, and lactate production. Thus, human malignancies with high TRAP1 expression, and CRCs among others, are characterized by a predominant glycolytic metabolism [15, 16, and this study] whereas human tumors with TRAP1 downregulation show mostly an oxidative metabolism [14]. Mechanistically, TRAP1 regulation of glycolysis relies primarily on its capacity to interact with PFK1 likely on the ER and enable PFK1 glycolytic activity preventing its ubiquitination/degradation (see the Graphical Abstract). This mechanism is consistent with previous observations by our group showing that TRAP1 is associated with the ER membranes, facing the cytosol [24] and plays a cotranslational quality control on a network of client proteins (i.e., sorcin, F1ATPase, BRAF, CDK1) which yields enhancement of their expression and lowers their proteasomal degradation [9]. This TRAP1 function is relevant for its regulation of specific cell functions and for its role in tumor progression. In such a view, TRAP1 enhancement of Warburg metabolism is likely crucial in supplying cancer cells with metabolic substrates and energetic molecules to support biosynthetic processes and cell proliferation.

It is intriguing that TRAP1 regulation of PFK1 activity is finalized to maximize Warburg metabolism in response to the downregulation of mitochondrial respiration. Conditions of poor mitochondrial respiration (i.e., lack of pyruvate or inhibition of pyruvate uptake by mitochondria, which prevents its entry in TCA cycle) [46] maximize TRAP1 capacity to enhance PFK1 activity and lactate production. Consistently, supplementation with pyruvate, which drives acetyl‐CoA production, enters in the TCA cycle and enhances mitochondrial respiration [47], results in loss of TRAP1 interaction with PFK1 and parallel lowering of PFK1 activity and glycolytic metabolism. Mechanistically, this interplay is dependent on both TRAP1 expression, which prevents PFK1 ubiquitination/degradation, and levels of citrate, a metabolic product of TCA cycle and an allosteric inhibitor of PKF1 [37, 48]. Indeed, citrate levels are higher in cell supplemented with pyruvate and/or silenced for TRAP1, both conditions characterized by an enhancement of OXPHOS. Consistently, cell supplementation with citrate, mimicking its enhanced production by mitochondrial respiratory chain, results in downregulation of PFK1 activity and loss of TRAP1 control of glycolysis. Thus, TRAP1 favors Warburg metabolism by maximizing PKF1 activity/stability to balance the downregulation of mitochondrial respiration. Remarkably, this mechanism is likely used to regulate tumor metabolic state depending on energetic requirements and extracellular stresses (see the Graphical Abstract).

Clinically relevant is the observation that high glycolytic metabolism results in poorer response to cetuximab in RAS‐wild‐type mCRCs, spheroids, and cell lines. Indeed, PFK1 expression and lactate production are under the control of EGFR signaling in CRC cells (this study) as previously observed in head and neck carcinoma cells [49]. Furthermore, TRAP1 upregulation and the parallel enhancement of glycolytic metabolism correlate with lower response to EGFR inhibition in cetuximab‐sensitive CRC cells. The inhibition of glycolytic metabolism or TRAP1 knock down in combination with cetuximab results in downregulation of lactate production and, interestingly, improved cetuximab cytostatic activity in patient‐derived spheres and immortalized cell lines. Finally, high ^18^F‐FDG PET uptake correlates with poor/lack of response to cetuximab first‐line combination chemotherapy in RAS‐wild‐type mCRCs. Thus, CRC cells may use metabolic rewiring to escape EGFR inhibition and this may account for poor response to EGFR inhibitors in a subgroup of human RAS‐wild‐type mCRCs [50]. Indeed, much evidence supports the role of metabolic plasticity of cancer cells in drug resistance [51]. We previously observed that metabolic rewiring toward mitochondrial respiration drives resistance to 5‐fluorouracil (FU) in FU‐resistant CRC cells [52] and to platinum in ovarian carcinoma cells upon TRAP1 downregulation [14]. More recent observations suggest that metabolic remodeling toward glycolysis favors resistance to EGFR inhibitors in EGFR‐mutated lung carcinoma [38]. As suggested/investigated by other groups, metabolic pathways represent novel molecular targets to counteract drug resistance and cancer progression. In such a view, TRAP1 protein network deserves to be studied as predictive biomarker of poor response to EGFR monoclonals in RAS‐wild‐type human CRCs and novel putative TRAP1 inhibitors [9, 53] warrant to be tested to boost the activity of EGFR monoclonals in human CRCs with predominant/preferential Warburg metabolism.

## 5 Conclusions

This study provides the proof of concept that TRAP1 is a determinant of metabolic rewiring in human CRCs by the modulation of PFK1 activity/stability and favors resistance to EGFR inhibitors through the regulation of glycolytic metabolism.

## Conflict of interest

The authors declare no conflict of interest.

## Author contributions

FM, ML, and FE involved in study concept and design. VC, DSW, CP, RS, GL, VLB, MP, FC, AP, GS, and NC contributed to acquisition of data. FM, ML, FE, NC, and GS analyzed and interpreted the data FM, ML, and FE involved in critical revision of the manuscript; FM, ML, and FE supervised the study.

## Supporting information


**Fig. S1.** GLUT1 expression correlates with ^18^F‐FDG uptake in human colorectal carcinomas. Scatter plot representing the statistical correlation between SUV upon ^18^F‐FDG PET scan and GLUT1 expression in the cohort of 26 human CRCs.

**Fig. S2.** TRAP1 regulates glycolytic metabolism in human CRC cell lines. A. Row data of experiments reported in Figure 2A. TRAP1 immunoblot analysis and corresponding lactate production and OCR data derived from independent siTRAP1 preparations. B. Relative lactate production and 2‐DG uptake in transient TRAP1‐silenced HCT116 cells. Inserts: TRAP1, GLUT1 and MCT4 immunoblot analysis in transient TRAP1‐silenced HCT116 cells. C‐D. Relative glucose uptake, lactate production (C) and OCR (D) in TRAP1‐silenced HCT116 cells cultured in the presence of high (16.6 mM) or low (5.5 mM) glucose concentration. C. Inserts: TRAP1 immunoblot analysis in TRAP1‐silenced HCT116 cells cultured in high or low glucose. The *Mann Whitney* test was used to establish the statistical significance between two group (p<0.05). C. *Anova test*: p=0.0001; Bonferroni post hoc test: siNEG high glucose vs siTRAP1 high glucose, lactate production: *p<0.0001*, glucose uptake: *p<0.0001;* siNEG low glucose vs siTRAP1 low glucose, lactate production: *p<0.01*, glucose uptake: *p<0.05*).

**Fig. S3.** TRAP1 regulation of glycolysis is independent from BRAF quality control. A‐B. Relative 2DG uptake and lactate production in HT29 cells transiently silenced for BRAF and transfected with TRAP1 cDNA (A) or in HCT116 cells transiently silenced for TRAP1 and transfected with the BRAFV600E mutant (B). Inserts: TRAP1 and BRAF immunoblot analysis in HT29 cells silenced for BRAF and transfected with TRAP1 cDNA (A) or in HCT116 cells silenced for TRAP1 and transfected with the BRAFV600E mutant (B). The *Mann Whitney* test was used to establish the statistical significance between two group (p<0.05). A. *Anova test*: p=0.0057; Bonferroni post hoc test: siNEG pmock vs siBRAF pmock, glucose uptake: *p<0.001*, lactate production: *p<0.001;* siNEG pmock vs siNEG pTRAP1, glucose uptake: *p<0.001,* lactate production: *p<0.001*; siNEG pTRAP1 vs siBRAF pTRAP1: glucose uptake: *p>0.05,* lactate production*: p>0.05*. B. *Anova test*: p<0.0001; Bonferroni post hoc test: siNEG pmock vs siTRAP1 pmock, glucose uptake: *p<0.001*, lactate production: *p<0.001;* siNEG pmock vs siNEG pBRAF, glucose uptake: *p<0.001*, lactate production*: p<0.001*; siNEG pBRAF vs siTRAP1 pBRAF: glucose uptake: *p<0.001*, lactate production: *p<0.001*.

**Fig. S4.** TRAP1 regulates glycolysis through PFK1. A. PFK1 and TRAP1 immunoblot analysis in shTRAP1 HCT116 cells. B. Relative PFK1 activity in TRAP1‐silenced HCT116 cells cultured in the presence of high (16.6 mM) or low (5.5 mM) glucose concentration. Inserts: TRAP1 and PFK1 immunoblot analysis in TRAP1‐silenced HCT116 cells cultured in high or low glucose. C. Real Time PCR analysis of PFK1 mRNA expression in TRAP1‐silenced HCT116 cells. Insert: PFK1 and TRAP1 immunoblot analysis in TRAP1‐silenced HCT116 cells. D. Relative PFK1 activity and lactate production in shTRAP1 HCT116 cells transfected with TRAP1 cDNA. Insert: PFK1 and TRAP1 immunoblot analysis in shTRAP1 HCT116 cells transfected with TRAP1 cDNA. The *Mann Whitney* test was used to establish the statistical significance between two group (p<0.05). B. *Anova test*: p=0.0001; Bonferroni post hoc test: siNEG high glucose vs siTRAP1 high glucose: *p<0.0001;* siNEG low glucose vs siTRAP1 low glucose: *p<0.01*. D. *Anova test*: p=0.001; Bonferroni post hoc test: scramble pmock vs shTRAP1 pmock, lactate production: *p<0.001*, PFK1 activity: *p< 0.001;* shTRAP1 pmock vs shTRAP1 pTRAP1 lactate production: *p<0.01*, PFK1 activity: *p< 0.01*.

**Fig. S5.** ER‐associated TRAP1 is responsible for regulation of glycolytic metabolism. A‐B. TRAP1 immunoblot analysis in cytosolic, ER and mitochondrial subcellular fractions of TRAP‐silenced HCT116 cells (A) and TRAP‐silenced HCT116 cells transfected with the Δ1‐59 TRAP1‐Myc deletion mutant lacking the mitochondrial targeting sequence (B). C. PFK1 activity and lactate production in shTRAP1 HCT116 cells transfected with pMock or the Δ1‐59 TRAP1‐Myc deletion mutant. Insert. TRAP1 and PFK1 immunoblot analysis in shTRAP1 HCT116 cells transfected with pMock or the Δ1‐59 TRAP1‐Myc deletion mutant. The *Mann Whitney* test was used to establish the statistical significance between two group (p<0.05). C. *Anova test*: p=0.0001; Bonferroni post hoc test: scramble pmock vs shTRAP1 pmock, lactate production: *p<0.001*, PFK1 activity: *p<0.001*; scramble pmock vs scramble pΔ1‐59TRAP1, lactate production: *p<0.0001*, PFK1 activity: *p<0.0001*; scramble pΔ1‐59TRAP1 vs shTRAP1 pΔ1‐59TRAP1, lactate production: *p<0.001*, PFK1 activity: *p< 0.001*.

**Fig. S6.** PFK1 expression is regulated by EGFR signaling. A. PFK1, TRAP1 and MCT4 immunoblot analysis in CaCo2 cells incubated with 100‐500 nM cetuximab for 12 h.

**Fig. S7.** Inhibition of glycolytic metabolism or TRAP1 silencing results in attenuation of spheroid formation. Representative images of spheroid formation assay in RAS‐wild type patients‐derived CRC spheroids (SA54 cells) cultured in standard medium (A) or incubated with 250 nM cetuximab (B), 10 μM 2DG (C) or 10 μM 3PO (E) for 48 h or silenced for TRAP1 (G) or exposed to the combination of cetuximab and 2DG (D), 3PO (F) or TRAP1 silencing (H).

**Fig. S8.** High glycolytic metabolism and TRAP1 expression correlate with poor response to cetuximab. A‐B. Cell cycle distribution (A) and relative PFK1 activity and lactate production (B) in RAS‐wild type CaCo2 cells incubated with 250 nM cetuximab, 10 μM 2DG or 10 μM 3PO for 15 h or silenced for TRAP1 or exposed to the combination of cetuximab and 2DG, 3PO or TRAP1 silencing. Insert: PFK1 and TRAP1 immunoblot analysis in TRAP1‐silenced Caco2 cells. The *Mann Whitney* test was used to establish the statistical significance between two group (p<0.05).

**Table S1.** Baseline characteristics of colorectal carcinoma patients.

**Table S2.** TRAP1 and GLUT1 protein levels and ^18^F‐FDG uptake (SUVmax) in human colorectal carcinomas
