# Supplementary figures and images for: TRAP1 enhances Warburg metabolism through modulation of PFK1 expression/activity and favors resistance to EGFR inhibitors in human colorectal carcinomas
Source: Mol Oncol. 2020 Oct 30;14(12):3030–47. doi: 10.1002/1878-0261.12814 (PMC7718945; doi:10.1002/1878-0261.12814)

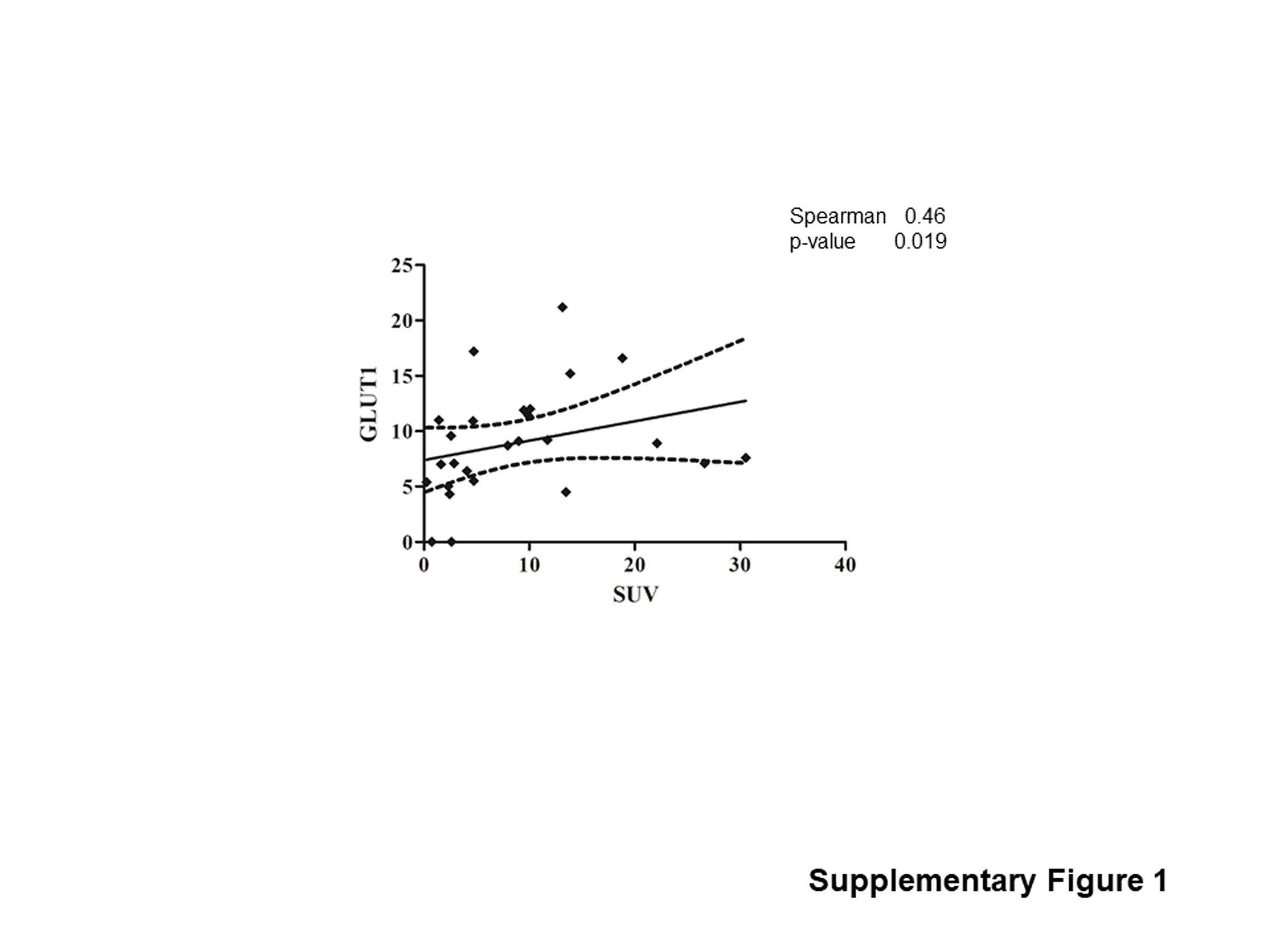

Supplement: Supplementary file 1 — Fig. S1. GLUT1 expression correlates with 18F‐FDG uptake in human colorectal carcinomas. Scatter plot representing the statistical correlation between SUV upon 18F‐FDG PET scan and GLUT1 expression in the cohort of 26 human CRCs. [file MOL2-14-3030-s001.tif]

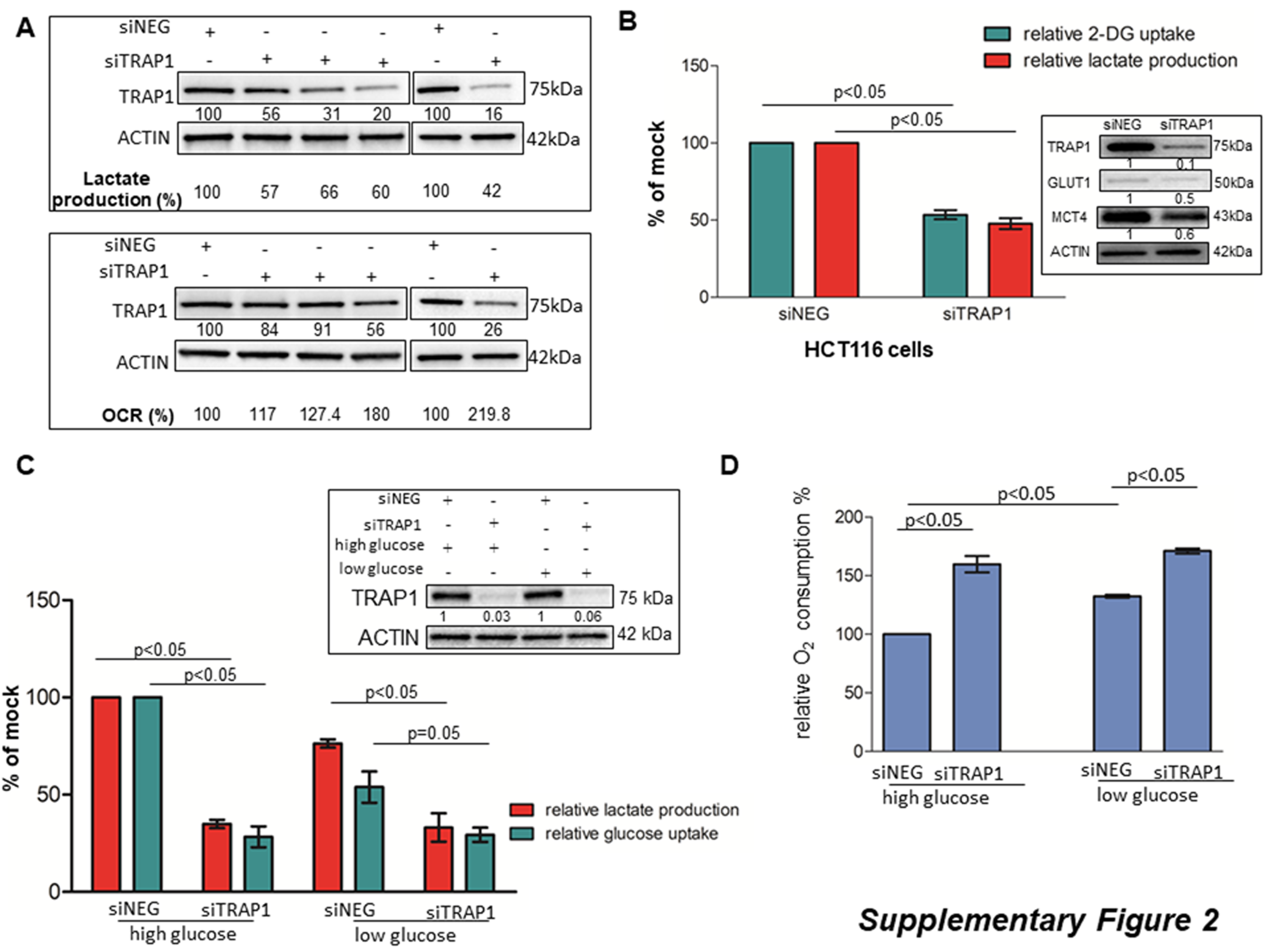

Supplement: Supplementary file 2 — Fig. S2. TRAP1 regulates glycolytic metabolism in human CRC cell lines. A. Row data of experiments reported in Figure 2A. TRAP1 immunoblot analysis and corresponding lactate production and OCR data derived from independent siTRAP1 preparations. B. Relative lactate production and 2‐DG uptake in transient TRAP1‐silenced HCT116 cells. Inserts: TRAP1, GLUT1 and MCT4 immunoblot analysis in transient TRAP1‐silenced HCT116 cells. C‐D. Relative glucose uptake, lactate production (C) and OCR (D) in TRAP1‐silenced HCT116 cells cultured in the presence of high (16.6 mM) or low (5.5 mM) glucose concentration. C. Inserts: TRAP1 immunoblot analysis in TRAP1‐silenced HCT116 cells cultured in high or low glucose. The Mann Whitney test was used to establish the statistical significance between two group (p<0.05). C. Anova test: p=0.0001; Bonferroni post hoc test: siNEG high glucose vs siTRAP1 high glucose, lactate production: p<0.0001, glucose uptake: p<0.0001; siNEG low glucose vs siTRAP1 low glucose, lactate production: p<0.01, glucose uptake: p<0.05). [file MOL2-14-3030-s002.tif]

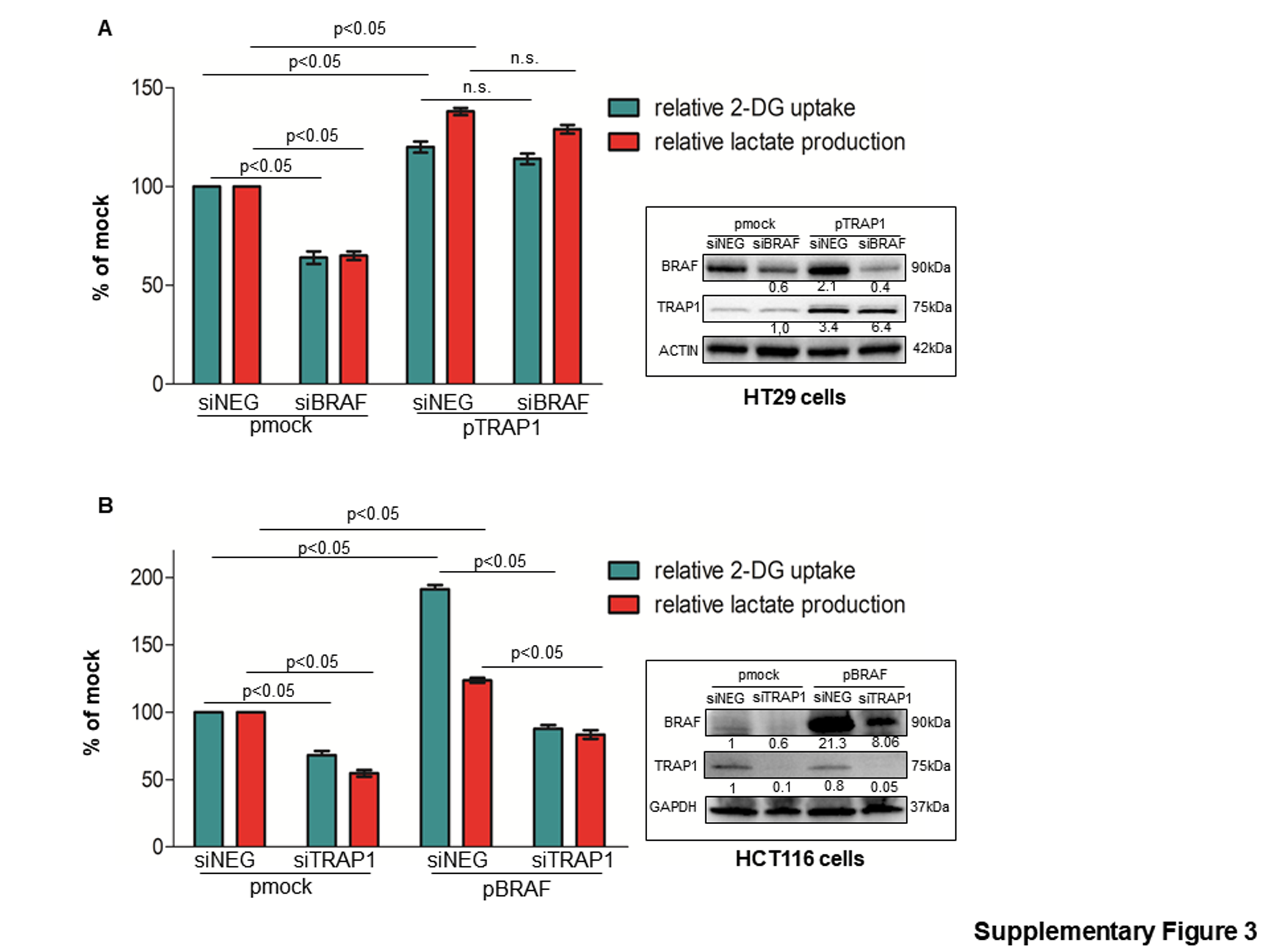

Supplement: Supplementary file 3 — Fig. S3. TRAP1 regulation of glycolysis is independent from BRAF quality control. A‐B. Relative 2DG uptake and lactate production in HT29 cells transiently silenced for BRAF and transfected with TRAP1 cDNA (A) or in HCT116 cells transiently silenced for TRAP1 and transfected with the BRAFV600E mutant (B). Inserts: TRAP1 and BRAF immunoblot analysis in HT29 cells silenced for BRAF and transfected with TRAP1 cDNA (A) or in HCT116 cells silenced for TRAP1 and transfected with the BRAFV600E mutant (B). The Mann Whitney test was used to establish the statistical significance between two group (p<0.05). A. Anova test: p=0.0057; Bonferroni post hoc test: siNEG pmock vs siBRAF pmock, glucose uptake: p<0.001, lactate production: p<0.001; siNEG pmock vs siNEG pTRAP1, glucose uptake: p<0.001, lactate production: p<0.001; siNEG pTRAP1 vs siBRAF pTRAP1: glucose uptake: p>0.05, lactate production: p>0.05. B. Anova test: p<0.0001; Bonferroni post hoc test: siNEG pmock vs siTRAP1 pmock, glucose uptake: p<0.001, lactate production: p<0.001; siNEG pmock vs siNEG pBRAF, glucose uptake: p<0.001, lactate production: p<0.001; siNEG pBRAF vs siTRAP1 pBRAF: glucose uptake: p<0.001, lactate production: p<0.001. [file MOL2-14-3030-s003.tif]

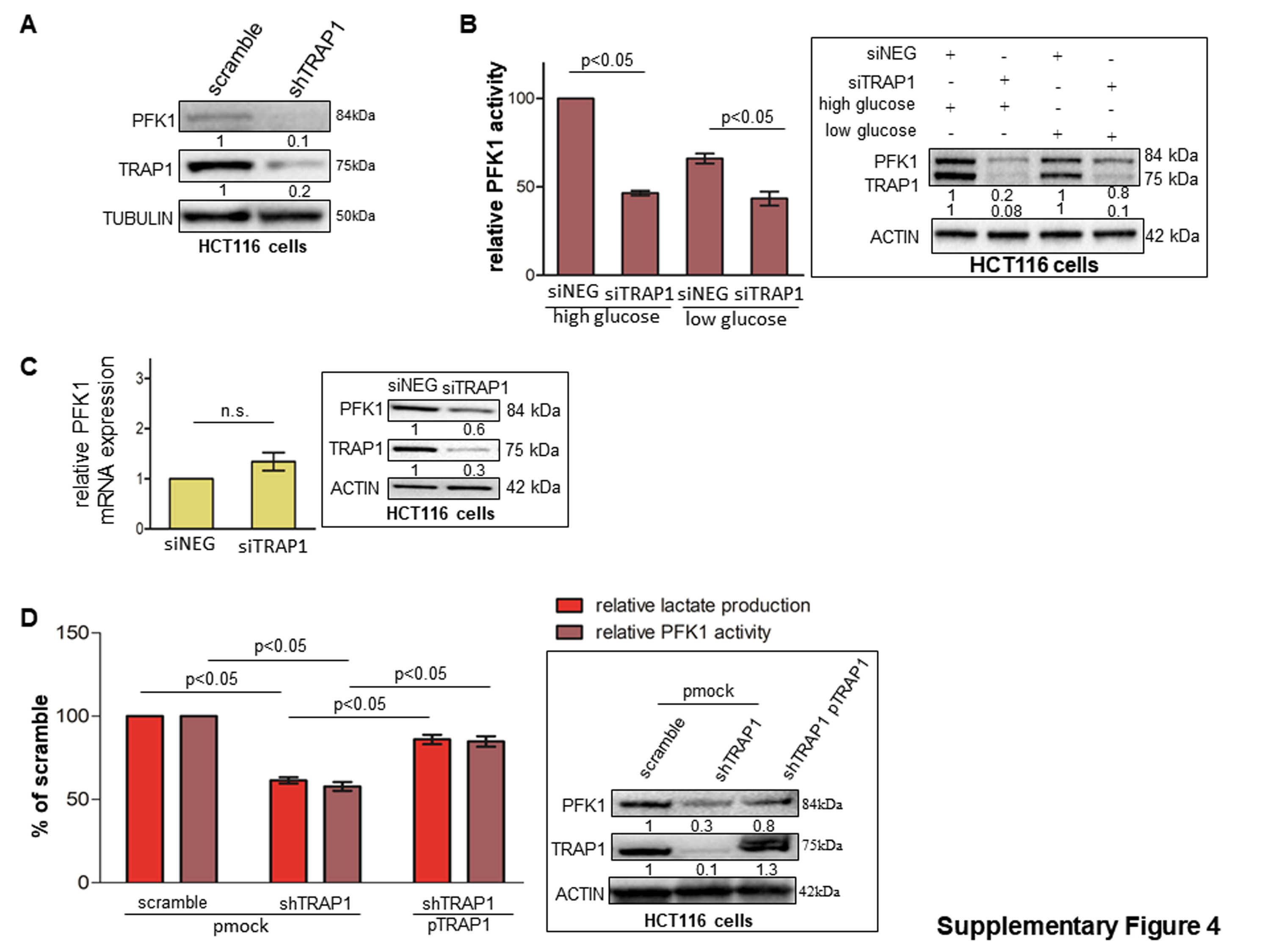

Supplement: Supplementary file 4 — Fig. S4. TRAP1 regulates glycolysis through PFK1. A. PFK1 and TRAP1 immunoblot analysis in shTRAP1 HCT116 cells. B. Relative PFK1 activity in TRAP1‐silenced HCT116 cells cultured in the presence of high (16.6 mM) or low (5.5 mM) glucose concentration. Inserts: TRAP1 and PFK1 immunoblot analysis in TRAP1‐silenced HCT116 cells cultured in high or low glucose. C. Real Time PCR analysis of PFK1 mRNA expression in TRAP1‐silenced HCT116 cells. Insert: PFK1 and TRAP1 immunoblot analysis in TRAP1‐silenced HCT116 cells. D. Relative PFK1 activity and lactate production in shTRAP1 HCT116 cells transfected with TRAP1 cDNA. Insert: PFK1 and TRAP1 immunoblot analysis in shTRAP1 HCT116 cells transfected with TRAP1 cDNA. The Mann Whitney test was used to establish the statistical significance between two group (p<0.05). B. Anova test: p=0.0001; Bonferroni post hoc test: siNEG high glucose vs siTRAP1 high glucose: p<0.0001; siNEG low glucose vs siTRAP1 low glucose: p<0.01. D. Anova test: p=0.001; Bonferroni post hoc test: scramble pmock vs shTRAP1 pmock, lactate production: p<0.001, PFK1 activity: p< 0.001; shTRAP1 pmock vs shTRAP1 pTRAP1 lactate production: p<0.01, PFK1 activity: p< 0.01. [file MOL2-14-3030-s004.tif]

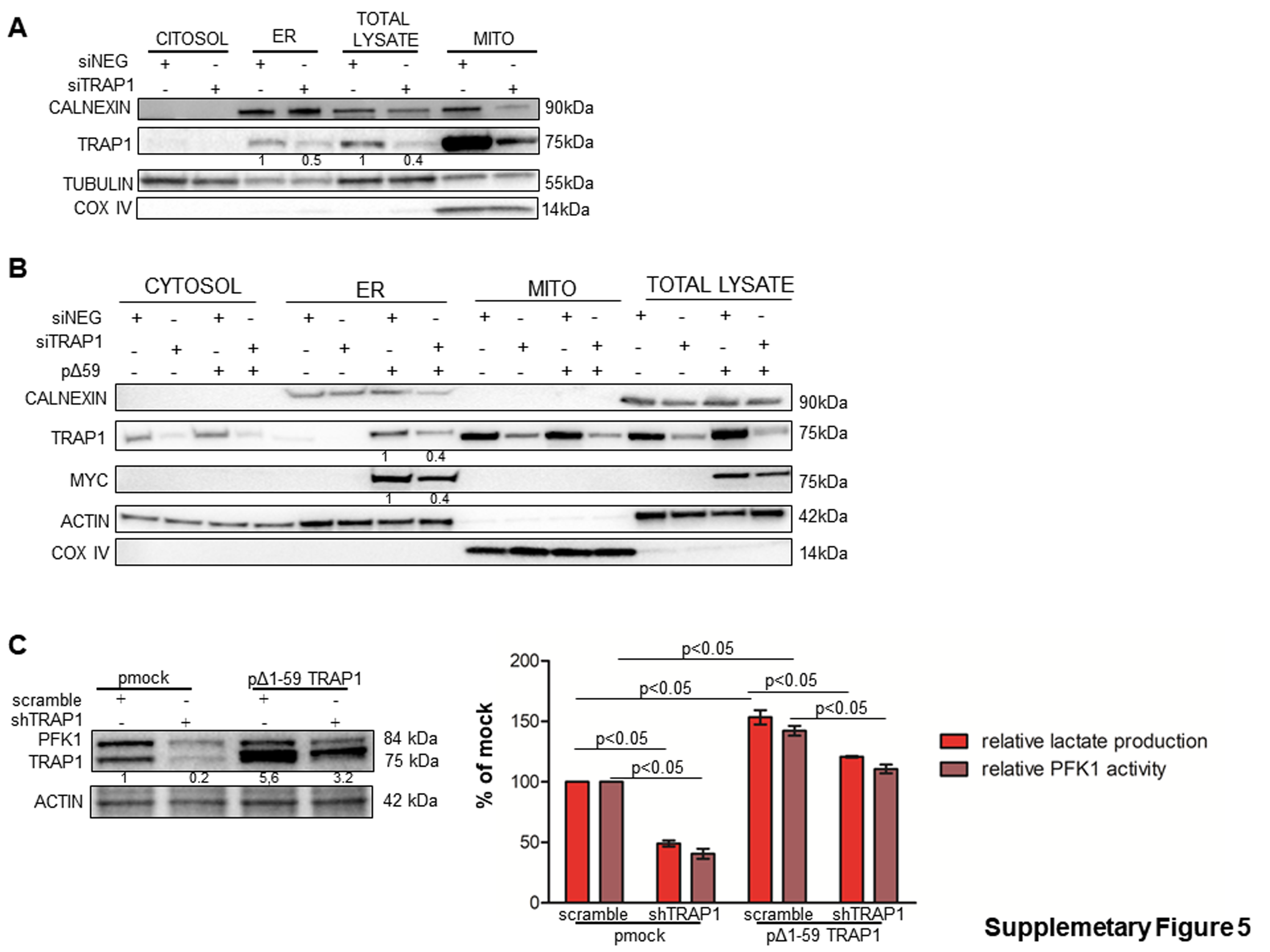

Supplement: Supplementary file 5 — Fig. S5. ER‐associated TRAP1 is responsible for regulation of glycolytic metabolism. A‐B. TRAP1 immunoblot analysis in cytosolic, ER and mitochondrial subcellular fractions of TRAP‐silenced HCT116 cells (A) and TRAP‐silenced HCT116 cells transfected with the Δ1‐59 TRAP1‐Myc deletion mutant lacking the mitochondrial targeting sequence (B). C. PFK1 activity and lactate production in shTRAP1 HCT116 cells transfected with pMock or the Δ1‐59 TRAP1‐Myc deletion mutant. Insert. TRAP1 and PFK1 immunoblot analysis in shTRAP1 HCT116 cells transfected with pMock or the Δ1‐59 TRAP1‐Myc deletion mutant. The Mann Whitney test was used to establish the statistical significance between two group (p<0.05). C. Anova test: p=0.0001; Bonferroni post hoc test: scramble pmock vs shTRAP1 pmock, lactate production: p<0.001, PFK1 activity: p<0.001; scramble pmock vs scramble pΔ1‐59TRAP1, lactate production: p<0.0001, PFK1 activity: p<0.0001; scramble pΔ1‐59TRAP1 vs shTRAP1 pΔ1‐59TRAP1, lactate production: p<0.001, PFK1 activity: p< 0.001. [file MOL2-14-3030-s005.tif]

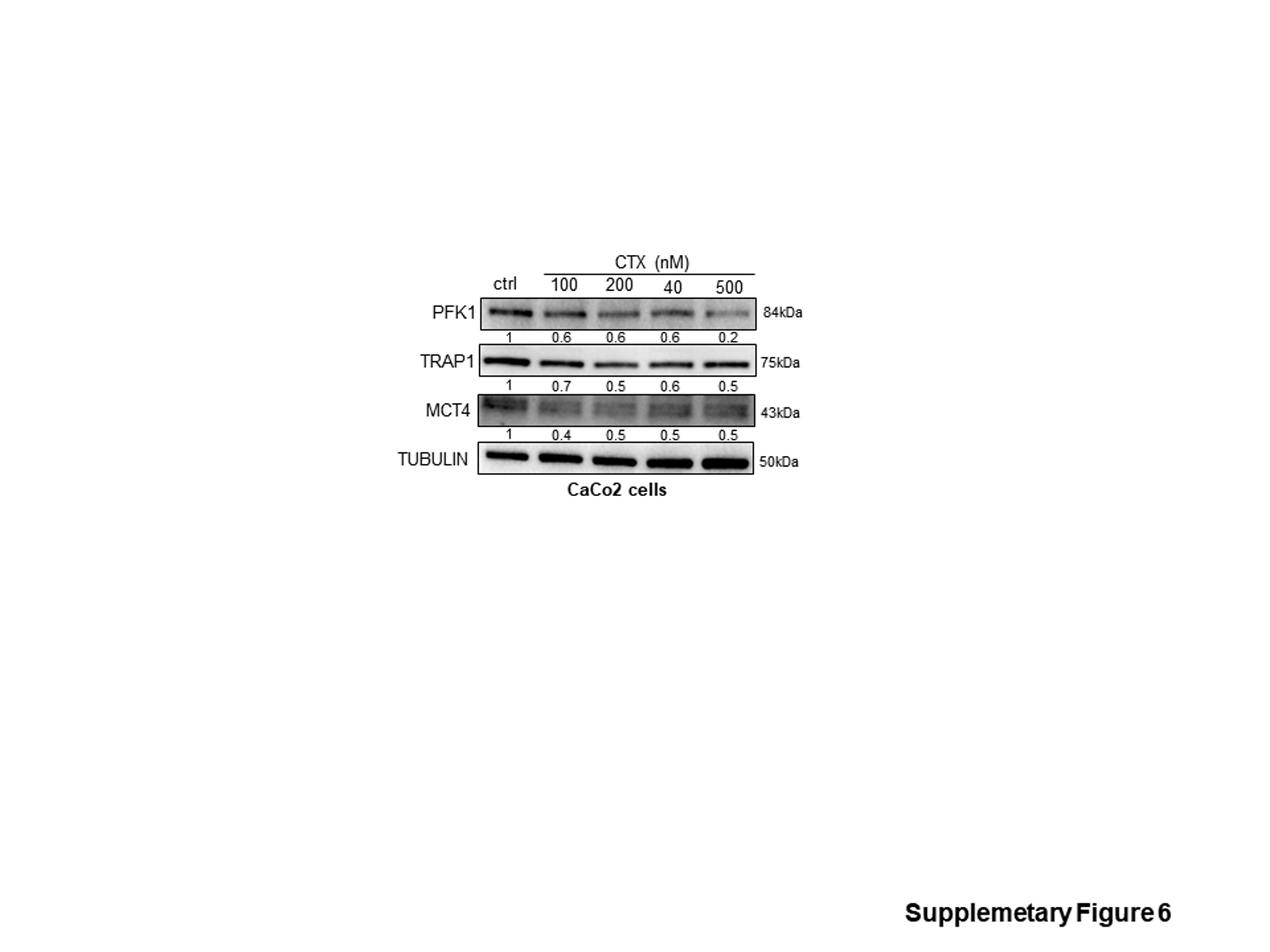

Supplement: Supplementary file 6 — Fig. S6. PFK1 expression is regulated by EGFR signaling. A. PFK1, TRAP1 and MCT4 immunoblot analysis in CaCo2 cells incubated with 100‐500 nM cetuximab for 12 h. [file MOL2-14-3030-s006.tif]

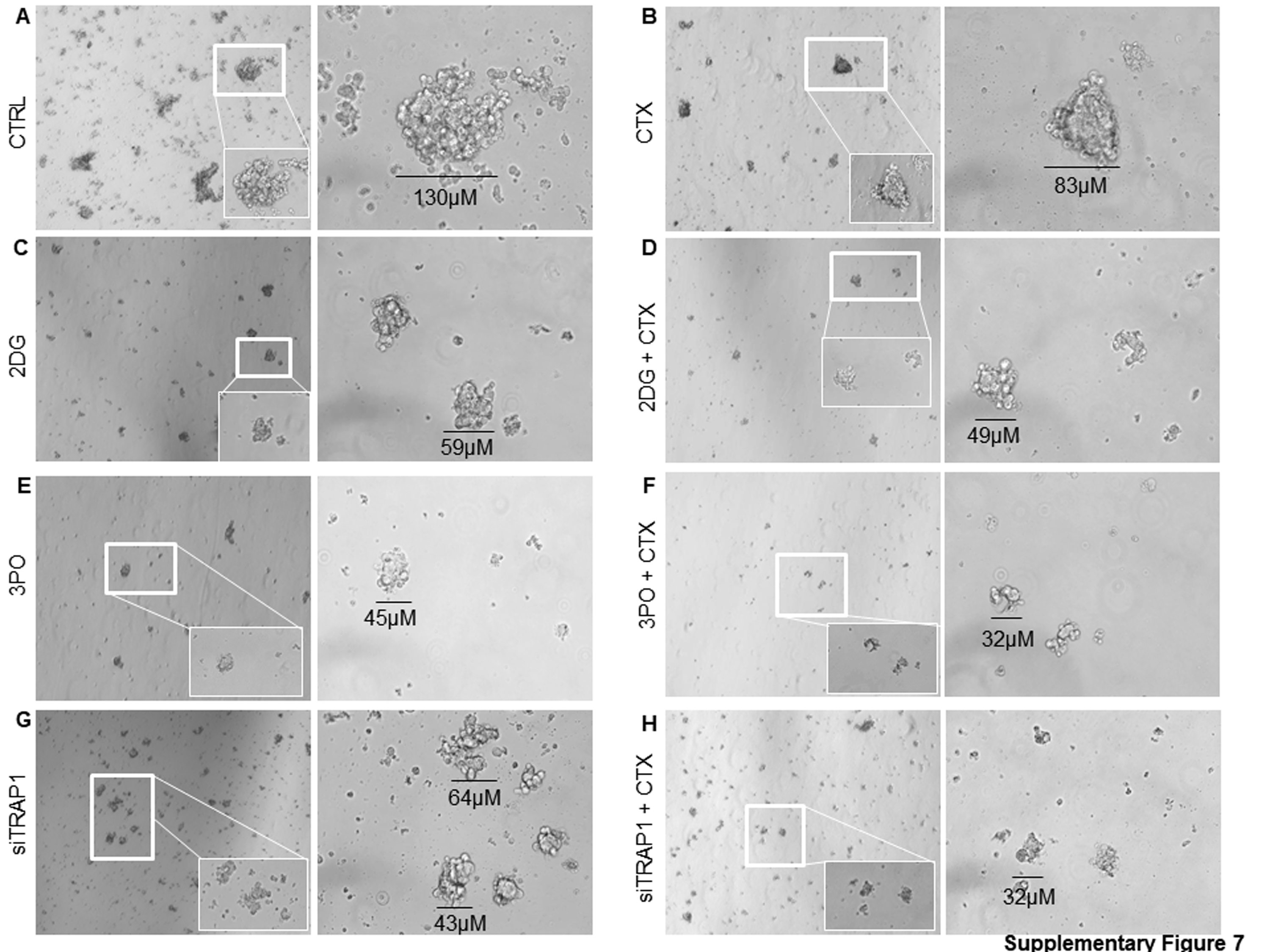

Supplement: Supplementary file 7 — Fig. S7. Inhibition of glycolytic metabolism or TRAP1 silencing results in attenuation of spheroid formation. Representative images of spheroid formation assay in RAS‐wild type patients‐derived CRC spheroids (SA54 cells) cultured in standard medium (A) or incubated with 250 nM cetuximab (B), 10 μM 2DG (C) or 10 μM 3PO (E) for 48 h or silenced for TRAP1 (G) or exposed to the combination of cetuximab and 2DG (D), 3PO (F) or TRAP1 silencing (H). [file MOL2-14-3030-s007.tif]

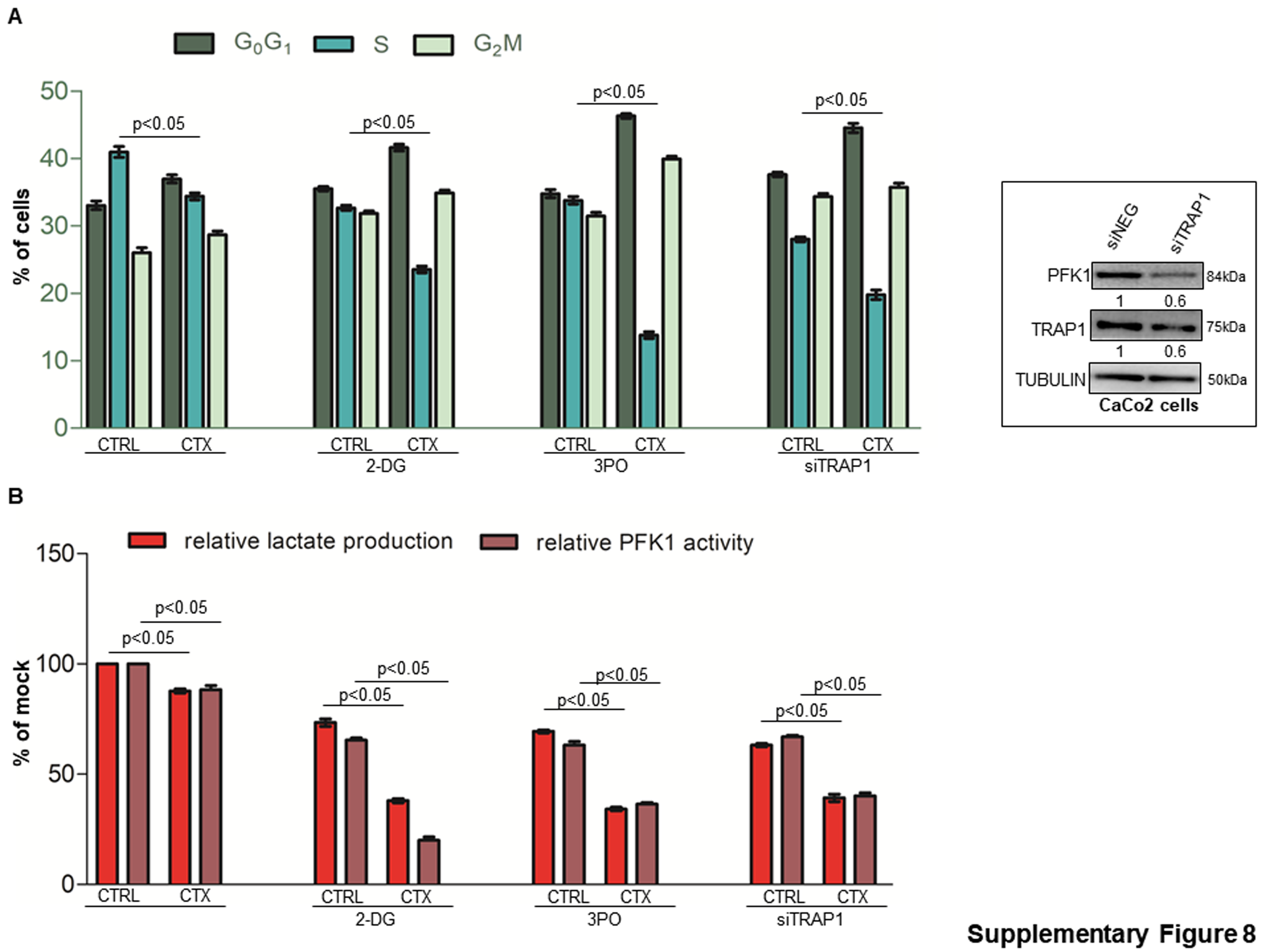

Supplement: Supplementary file 8 — Fig. S8. High glycolytic metabolism and TRAP1 expression correlate with poor response to cetuximab. A‐B. Cell cycle distribution (A) and relative PFK1 activity and lactate production (B) in RAS‐wild type CaCo2 cells incubated with 250 nM cetuximab, 10 μM 2DG or 10 μM 3PO for 15 h or silenced for TRAP1 or exposed to the combination of cetuximab and 2DG, 3PO or TRAP1 silencing. Insert: PFK1 and TRAP1 immunoblot analysis in TRAP1‐silenced Caco2 cells. The Mann Whitney test was used to establish the statistical significance between two group (p<0.05). [file MOL2-14-3030-s008.tif]
