# Supplementary material for: TRAP1 enhances Warburg metabolism through modulation of PFK1 expression/activity and favors resistance to EGFR inhibitors in human colorectal carcinomas
Source: Mol Oncol. 2020 Oct 30;14(12):3030–47. doi: 10.1002/1878-0261.12814 (PMC7718945; doi:10.1002/1878-0261.12814)
Supplement: Supplementary file 9 — Table S1. Baseline characteristics of colorectal carcinoma patients. [file MOL2-14-3030-s009.doc]

**Supplementary Table 1**. Baseline characteristics of colorectal carcinoma patients: Cohort 1 (n=26) and Cohort 2 (n= 15; RAS-wild type metastatic CRCs treated with first-line chemotherapy combined with cetuximab).

|  | **Cohort 1** | | **Cohort 2** | |
| --- | --- | --- | --- | --- |
|  | **n.** | **%** | **n.** | **%** |
| **Patients** | 26 |  | 15 |  |
| *Age* |  |  |  |  |
| **Median (years)**  **Range** | 71  45-90 |  | 64  30-85 |  |
| S*ex* |  |  |  |  |
| **Female** | 11 | 42 | 5 | 33 |
| **Males** | 15 | 58 | 10 | 67 |
|  |  |  |  |  |
| ***Tumor stage*** |  |  |  |  |
| **T2** | 3 | 12 | 0 | 0 |
| **T3** | 18 | 69 | 7 | 47 |
| **T4** | 5 | 19 | 8 | 53 |
|  |  |  |  |  |
| **Nx** | 1 | 4 | 2 | 13 |
| **N0** | 6 | 23 | 0 | 0 |
| **N1** | 9 | 35 | 3 | 20 |
| **N2** | 10 | 38 | 10 | 67 |
|  |  |  |  |  |
| **M0** | 14 | 54 | 0 | 0 |
| **M1** | 12 | 46 | 15 | 100 |
|  |  |  |  |  |
| ***Metastatic pattners*** |  |  |  |  |
| **Liver** | 11 | 58 | 10 | 36 |
| **Lung** | 3 | 16 | 7 | 25 |
| **Peritoneum** | 0 | 0 | 6 | 21 |
| **Other sites** | 5 | 26 | 5 | 18 |
|  |  |  |  |  |
| ***Target lesions*** |  |  | 25 |  |
| **Liver** |  |  | 14 | 56 |
| **Lung** |  |  | 8 | 32 |
| **Others** |  |  | 3 | 12 |
|  |  |  |  |  |
| ***First line Chemotherapy*** |  |  |  |  |
| **FOLFOX** |  |  | 11 | 73 |
| **FOLFIRI** |  |  | 4 | 27 |
