# Supplementary material for: TRAP1 enhances Warburg metabolism through modulation of PFK1 expression/activity and favors resistance to EGFR inhibitors in human colorectal carcinomas
Source: Mol Oncol. 2020 Oct 30;14(12):3030–47. doi: 10.1002/1878-0261.12814 (PMC7718945; doi:10.1002/1878-0261.12814)
Supplement: Supplementary file 10 — Table S2. TRAP1 and GLUT1 protein levels and 18F‐FDG uptake (SUVmax) in human colorectal carcinomas [file MOL2-14-3030-s010.doc]

**Supplementary Table 2.** TRAP1 and GLUT1 protein levels and ^18^F-FDG uptake (SUVmax) in 26 human colorectal carcinomas. TRAP1 and GLUT1 are expressed as time increase in tumor compared to correspondent non-infiltrated peritumoral mucosas.

| **Case n.** | **TRAP1**  **protein levels** | **GLUT1**  **protein levels** | **SUVmax** |
| --- | --- | --- | --- |
| 1. | 4.50 | 2.61 | 0 |
| 2. | 1.40 | 2.43 | 4.30 |
| 3. | 1.35 | 2.31 | 5.00 |
| 4. | 1.80 | 4.06 | 6.40 |
| 5. | 12.86 | 13.86 | 15.20 |
| 6. | 5.40 | 2.85 | 7.10 |
| 7. | 0.30 | 0.74 | 0 |
| 8. | 0.38 | 0.25 | 5.40 |
| 9. | 32.24 | 9.46 | 11.90 |
| 10. | 3.41 | 4.72 | 5.50 |
| 11. | 8.96 | 18.82 | 16.60 |
| 12. | 1.91 | 1.61 | 7.00 |
| 13. | 8.28 | 8.98 | 9.10 |
| 14. | 0.80 | 1.41 | 11.00 |
| 15. | 39.90 | 22.10 | 8.90 |
| 16. | 16.41 | 11.72 | 9.20 |
| 17. | 49.35 | 26.61 | 7.10 |
| 18. | 12.59 | 10.05 | 12.00 |
| 19. | 5.03 | 2.57 | 9.60 |
| 20. | 46.33 | 30.52 | 7.60 |
| 21. | 26.34 | 13.13 | 21.20 |
| 22. | 19.75 | 9.93 | 11.40 |
| 23. | 9.32 | 4.66 | 10.90 |
| 24. | 19.74 | 13.46 | 4.50 |
| 25. | 6.33 | 4.72 | 17.20 |
| 26. | 12.50 | 7.94 | 8.70 |
